# The effect of constitutive root isoprene emission on root phenotype and physiology under control and salt stress conditions

**DOI:** 10.1002/pld3.617

**Published:** 2024-07-06

**Authors:** Manuel Bellucci, Mohammad Golam Mostofa, Sarathi M. Weraduwage, Yuan Xu, Mostafa Abdelrahman, Laura De Gara, Francesco Loreto, Thomas D. Sharkey

**Affiliations:** ^1^ Department of Energy Plant Research Laboratory Michigan State University East Lansing Michigan USA; ^2^ Department of Science and Technology for Humans and the Environment Università Campus Bio‐Medico di Roma Rome Italy; ^3^ Plant Resilience Institute Michigan State University East Lansing Michigan USA; ^4^ Department of Biochemistry and Molecular Biology Michigan State University East Lansing Michigan USA; ^5^ Department of Biology and Biochemistry Bishop's University Sherbrooke Quebec Canada; ^6^ Institute of Genomics for Crop Abiotic Stress Tolerance Texas Tech University Lubbock Texas USA; ^7^ Department of Biology University of Naples Federico II Naples Italy; ^8^ Institute for Sustainable Plant Protection The National Research Council of Italy (CNR‐IPSP) Sesto Fiorentino (Florence) Italy

**Keywords:** cytokinins, isoprene synthase, methylerythritol 4‐phosphate (MEP) metabolites, root phenotype, salinity, transcriptomics

## Abstract

Isoprene, a volatile hydrocarbon, is typically emitted from the leaves of many plant species. Given its well‐known function in plant growth and defense aboveground, we examined its effects on root physiology. We used isoprene‐emitting (IE) lines and a non‐emitting (NE) line of Arabidopsis and investigated their performance by analyzing root phenotype, hormone levels, transcriptome, and metabolite profiles under both normal and salt stress conditions. We show that IE lines emitted tiny amounts of isoprene from roots and showed an increased root/shoot ratio compared with NE line. Isoprene emission exerted a noteworthy influence on hormone profiles related to plant growth and stress response, promoting root development and salt‐stress resistance. Methyl erythritol 4‐phosphate pathway metabolites, precursors of isoprene and hormones, were higher in the roots of IE lines than in the NE line. Transcriptome data indicated that the presence of isoprene increased the expression of key genes involved in hormone metabolism/signaling. Our findings reveal that constitutive root isoprene emission sustains root growth under saline conditions by regulating and/or priming hormone biosynthesis and signaling mechanisms and expression of key genes relevant to salt stress defense.

## 1 INTRODUCTION

Isoprene is a non‐methane biogenic volatile organic compound (BVOC) emitted by more than half of all terrestrial tropical species, and accounts for the largest flux of BVOCs from the biosphere to the atmosphere (Guenther et al., 2006) and well in excess of anthropogenic hydrocarbon fluxes to the atmosphere. Isoprene is synthesized from the plastidial methylerythritol 4‐phosphate (MEP) pathway product, dimethylallyl diphosphate (DMADP) in a reaction catalyzed by isoprene synthase (ISPS). Isoprene emission is metabolically expensive (14 NADPH and 20 ATP/isoprene) for plants (Sharkey & Yeh, 2001), although benefits may outweigh the cost, especially under the conditions like high temperature (Jardine et al., 2012) and oxidative stress (Vickers et al., 2009).

Isoprene is physiologically important to protect the photosynthetic apparatus of chloroplasts from the adverse effects of excessive heat and water‐shortage. The underlying proposed mechanisms include stabilization of thylakoid membranes, dissipation of excessive light energy, and protection against oxidative damage by quenching reactive oxygen species (ROS) (Loreto et al., 2001; Pollastri et al., 2019; Velikova et al., 2005). Because ROS accumulation is a common consequence of most abiotic stresses, the antioxidant roles of isoprene underlie a general defense mechanism against abiotic stresses (Loreto & Schnitzler, 2010). Recently, the hypothesis of a direct ROS‐quenching effect of isoprene has been revised. It is thought that isoprene effects on the transcriptome, proteome, and metabolome accounts for improved stress tolerance (Dani & Loreto, 2022; Lantz et al., 2019; Monson et al., 2021). Indeed, isoprene has a hormone‐like activity (Pollastri et al., 2021), acting as a signal molecule for regulating gene expression and biosynthesis and signaling cascades of several plant hormones, including cytokinins (CKs), jasmonic acid (JA), and salicylic acid (SA) (Dani et al., 2022; Dani & Loreto, 2022; Weraduwage et al., 2023; Zuo et al., 2019). The control of isoprene over specific metabolite production and defense mechanisms indicates the roles of isoprene in growth‐defense tradeoffs (Frank et al., 2021; Monson et al., 2020; Xu et al., 2020; Zuo et al., 2019). Isoprene is also known to modulate ROS‐mediated cellular signaling for reshaping plant developmental processes (Miloradovic van Doorn et al., 2020).

Isoprene studies have principally focused on the aboveground plant parts (either leaves or whole canopies), while the role of isoprene belowground, especially in the root system, has been rarely investigated. Root isoprene emission was observed in poplar (*Populus x canescens*) (Ghirardo et al., 2011; Miloradovic van Doorn et al., 2020) and in transgenic *Arabidopsis thaliana* harboring the *ISPS* of *Populus x canescens* (*PcISPS*, Loivamäki et al., 2007). The constitutive promoter of *PcISPS* is also active in the roots, particularly in the tips of the fine roots of poplar (Cinege et al., 2009; Miloradovic van Doorn et al., 2020). An upregulation of root growth‐related gene *phosphatidylinositol‐4‐phosphate‐5‐kinase 2* (*PIP5K2*), *transcription factor* (*MYB59*), and *nitrate transporter* (*NRT1.1*) was found in unstressed isoprene‐emitting (IE) leaves of *Arabidopsis* (Zuo et al., 2019). In poplar, the IE line showed a reduction in lateral root (LR) growth but a development of deeper root phenotype when compared with RNAi‐lines deficient in isoprene synthesis (Miloradovic van Doorn et al., 2020). These results clearly indicate isoprene connection to plant roots; however, it remains unknown whether plants could use this trait as an advantage to moderate stress resilience as observed in case of other BVOCs emitted from roots (Arimura, 2021; Asensio et al., 2012; Kigathi et al., 2019).

Roots are the anchorage system of plants and are essential for the uptake of mineral nutrients critical for plant growth and productivity. Roots are exposed to several abiotic stresses, such as water‐shortage and excess or deficiency of mineral nutrients, and adapt their architecture accordingly (Karlova et al., 2021). Likewise, roots are the primary plant organs that can promptly sense the abnormal accumulation of salts in the soils and initiate signals throughout the plant (Galvan‐Ampudia & Testerink, 2011). Salt accumulation is particularly deleterious for plant health in dry climates and is plaguing increasing areas of cultivable lands worldwide because of climate instability (Corwin, 2021; Minhas et al., 2020). In response to salt stress, roots can readjust their growth, dynamics, and architecture and mount defense mechanisms to overcome adverse consequences (Zou et al., 2022). This often requires signaling events to reprogram the architecture through gene‐to‐metabolite networks, resulting in avoidance and/or heightened protection against salt stress effects (Julkowska et al., 2017).

Recent genetic studies using different plant systems, including tobacco (*Nicotiana tabacum*), poplars (*Populus* spp.), and *Arabidopsis* report compelling evidence for isoprene signaling roles in enhancing plant defense mechanisms (Dani et al., 2022; Miloradovic van Doorn et al., 2020; Zuo et al., 2019). Several studies also indicate that isoprene emission from foliar organs play vital roles in plant physiological responses under various abiotic stresses (Jardine et al., 2012; Loreto & Schnitzler, 2010; Vickers et al., 2009). However, little is known about isoprene emission of roots, and its consequences on root physiology. Moreover, it is currently unknown whether root isoprene emission is regulated under any environmental stress, and if root isoprene emission has any effect on root metabolomes and transcriptomes.

In the current study, we used empty‐vector (EV) and wild‐type (WT) (both non‐emitters, NE), and isoprene emitting (IE) transgenic *Arabidopsis* lines (B2 and C4) to test root isoprene effects when exposing plants to salt stress. We investigated physiological responses of roots and the levels of phytohormones and stress metabolites, as well as the changes in MEP pathway metabolites in IE and NE plants under normal and saline conditions. We also examined the transcriptome levels by performing RNA‐sequencing (RNA‐seq) in the roots of IE and NE lines in the presence and absence of salt stress. We show that induction of isoprene emission promotes primary root (PR) growth, altering root metabolome (hormone, MEP metabolites, and amino acids) and transcriptome, under control and salt stress conditions.

## 2 RESULTS

### 2.1 Root isoprene emission, root growth, and biomass accumulation

**FIGURE 1:**
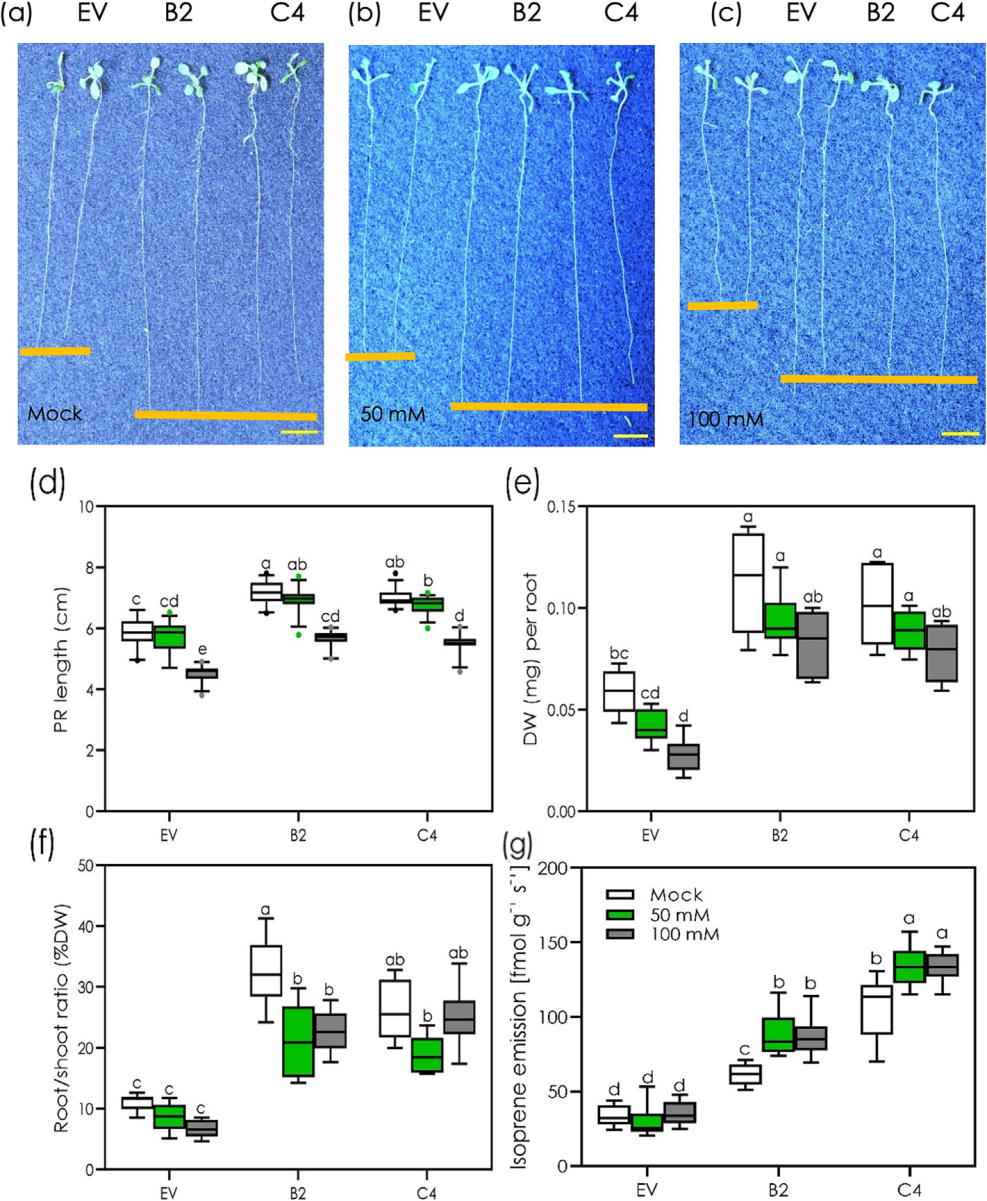
Root phenotypes, growth parameters and isoprene emission of non‐emitting (EV) and emitting (B2 and C4) Arabidopsis lines under control and salt‐stressed conditions. Representative root phenotype images (a–c), primary root (PR) length (d), dry weight (e), root/shoot ratio (f) and isoprene emission (g) of EV, B2, and C4 were recorded after exposure to 0 (mock), 50, and 100 mM of NaCl for 5 days (*n* = 48 plants). The horizontal line within the box corresponds to the median and the box marks the lower and upper quartiles. All data are means ± standard deviations. Green and gray points indicated outlier data. Different letters above the box plots indicate significant differences (*p* < .05) among the treatments and genotypes, based on two‐way ANOVA and Tukey's test. Scale bar = 1 cm.

Isoprene‐emitting B2 and C4 *A. thaliana* lines developed a deeper root phenotype, showing a higher PR growth under control conditions than the EV NE line (Figure 1a–d). This also occurred in soil, where 2‐week‐old IE lines showed a 30% higher PR growth with respect to NE (EV) roots (Figure S1).

Mild salt stress (50 mM NaCl) did not reduce PR length in both EV NE and IE lines, while severe salt stress (100 mM NaCl) resulted in a 30% and 20% reduction of PR length in EV NE and IE lines, respectively (Figure S2a).

IE lines showed significantly higher root biomass under both normal and salt stress conditions with respect to EV NE (Figure 1e). At 50 mM NaCl, the root biomass of EV NE and IE lines did not change compared with their respective control conditions (Figure 1e). Under severe salt stress, root DW was significantly reduced by 60% in the EV NE line, but only by 30% and 25% in the IE lines B2 and C4, respectively, compared with control conditions (Figure 1e; Figure S2b). IE lines also showed higher root/shoot ratio than EV NE, in control and under mild and severe salt stress conditions (Figure 1f), while no significant changes appeared in the shoot biomass between EV NE (slightly higher) and IE (Figure S3a), in contrast to that seen by Zuo et al. (2019).

A measurable level of constitutive isoprene emission was recorded in the roots of the IE lines B2 and C4, whereas isoprene emission was barely detectable in the EV line (Figure 1g). Salt stress affected the emission of isoprene by increasing its emission by 30% and 20%, respectively in B2 and C4 lines. Constitutive isoprene emission did not alter the number of LR in roots of B2 and C4 lines (Figure S3b).

Isoprene emission affected root gravitropic response in IE lines (Figure S4). When rotating the plates of unstressed 5‐day‐old seedlings by 90°, IE lines responded significantly more than EV line at 2, 5, and 7 h after rotation, which resulted in a steeper phenotype in B2 and C4 lines 24 h after plate rotation (Figure S4).

### 2.2 Pyruvate and MEP pathway metabolites

**FIGURE 2:**
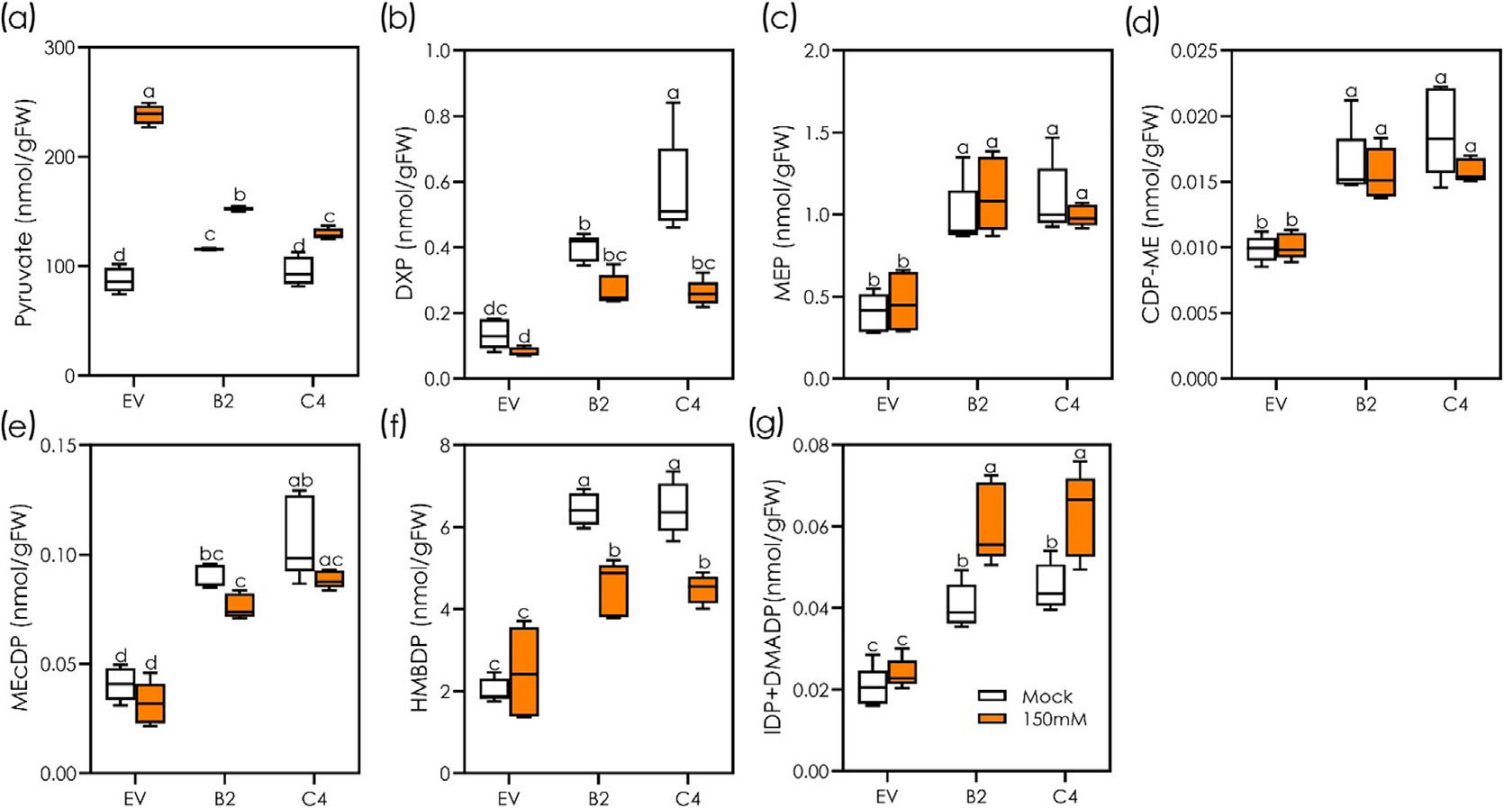
Levels of MEP metabolites in the roots of non‐emitting (EV) and emitting (B2 and C4) Arabidopsis lines under control and salt‐stressed conditions. Ten‐day‐old EV, B2, and C4 plants were exposed to 0 (mock) and 150 mM NaCl solutions for 4 h and the levels of (a) pyruvate, (b) 1‐deoxy‐d‐xylulose 5‐phosphate (DXP), (c) 2‐C‐methylerythritol 4‐phosphate (MEP), (d) 4‐diphosphocytidyl‐2‐C‐methylerythritol (CDP‐ME), (e) 2‐C‐methyl‐d‐erythritol 2,4‐cyclodiphosphate (MEcDP), (f) (E)‐4‐Hydroxy‐3‐methyl‐but‐2‐enyl diphosphate (HMBDP), (g) isopentenyl diphosphate (IDP) and dimethylallyl diphosphate (DMADP) were recorded (*n* = 4). The horizontal line within the box corresponds to the median and the box marks the lower and upper quartiles. All data are means *±* standard deviations. Different letters above the box plots indicate significant differences (*p* < .05) among the treatments and genotypes, based on two‐way ANOVA and Tukey's test.

Salinity caused a significant increase in the level of pyruvate in the EV line when compared with control condition. The increase of pyruvate level in the salt‐stressed IE lines was much smaller compared with EV but was significantly higher than in non‐stressed controls (Figure 2a). IE lines consistently maintained a higher level of MEP pathway metabolites compared with the NE EV line under both control and salt‐stressed conditions (Figure 2b–g). In particular, the levels of DXP, MEP, CDP‐ME, MEcDP, HMBDP, and IDP + DMADP were roughly two times higher in IE lines than in EV under control conditions. HMBDP decreased in salt‐stressed IE lines only, while slightly increasing (15%) in EV (Figure 2f). IDP + DMADP significantly increased in salt‐stressed IE lines compared with control conditions (Figure 2g).

### 2.3 Hormones

**FIGURE 3:**
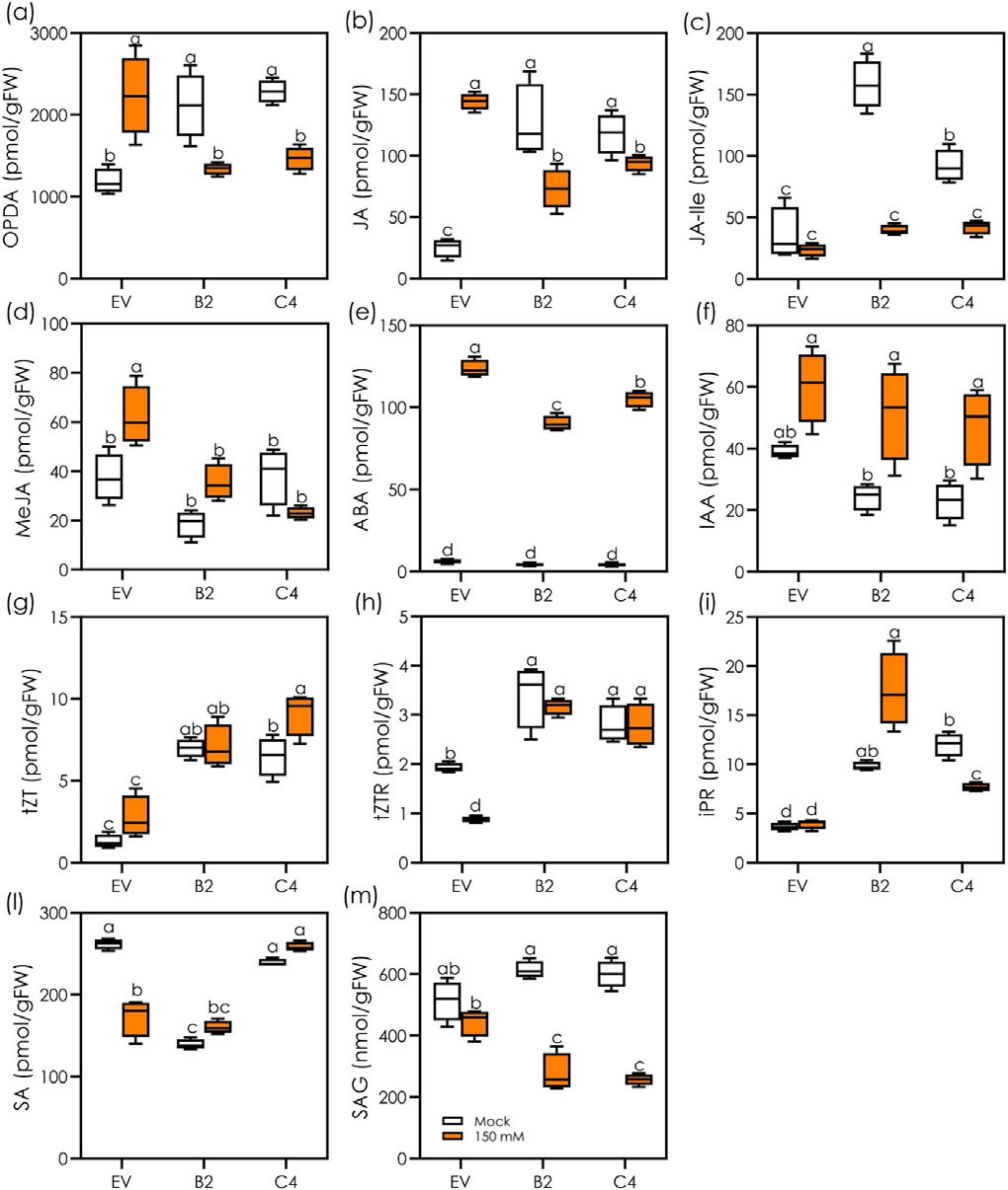
Effects of root isoprene emission on hormone levels in the roots of non‐emitting (EV) and emitting (B2 and C4) Arabidopsis lines under control and salt‐stressed conditions. The levels of different hormones and hormonal precursors, including (a) oxylipin 12‐oxo‐phytodienoic acid (OPDA), (b) jasmonic acid (JA), (c) JA‐isoleucine (JA‐Ile), (d) methyl‐jasmonate (MeJA), (e) abscisic acid (ABA), (f) indole acetic acid (IAA), (g) trans‐zeatin (tZT), (h) trans‐zeatin riboside (tZR), (i) isopentenyl riboside (iPR), (l) salicylic acid (SA), and (m) salicylic acid glucoside (SAG) were determined in root tissues of EV, B2, and C4 lines after exposure to 0 (mock) and 150 mM NaCl solution for 4 h (*n* = 4). The horizonal line within the box corresponds to the median and the box marks the lower and upper quartiles. All data are means ± standard deviations. Different letters above the box plots indicate the significance differences (P < .05) among the treatments and genotypes, based on two‐way ANOVA and Tukey's test.

When compared with the EV line, IE lines showed a significant increase in the levels of OPDA, JA, and JA‐Ile while exhibiting a similar level of MeJA under control conditions (Figure 3a–d). In response to salt stress, EV displayed a significant increase in the levels of OPDA, JA, and MeJA but a non‐significant change in JA‐Ile level when compared with control conditions (Figure 3a–d). On the other hand, the levels of JA, OPDA, and JA‐Ile but not MeJA significantly decreased in salt‐stressed IE B2 and C4 lines relative to control conditions (Figure 3a–d). The levels of ABA were constitutively very low in both NE and IE lines under control conditions (Figure 3e). Salt stress resulted in a significant increase in the ABA level by 30, 20 and 25 times in EV, B2, and C4 lines, respectively, in comparison with control conditions. IAA levels did not differ significantly among the lines in control conditions (Figure 3f). However, IAA levels increased significantly in salt‐stressed IE lines but to a non‐significant degree in the NE line (Figure 3f).

IE lines maintained a constantly higher level of cytokinins, including tZT, tZTR, and iPR, than the EV line under both control and salt stress conditions (Figure 3g–i). In the presence of salt stress, tZTR level significantly decreased (Figure 3h) whereas the levels of tZT and iPR remained at the control level in EV plants (Figure 3g,i). On the other hand, salt stress resulted in a significant increase of tZT and decrease of iPR levels in the C4 line when compared with that under normal condition. However, in B2 line, all the examined cytokinins remained at the control levels in response to salt stress (Figure 3g–i).

Without salt stress, SA level was comparable in EV and C4 lines, but significantly lower in the IE B2 line (Figure 3l). Salt stress severely decreased the level of SA (by 35%) in the EV line but had no significant effect on the level of SA in IE lines (Figure 3l). The level of SAG was comparable among the lines under control conditions (Figure 3m). However, salt treatment caused a significant decline of SAG in IE lines but not in the EV line when compared with control conditions (Figure 3m).

### 2.4 Amino acids and organic acids

**FIGURE 4:**
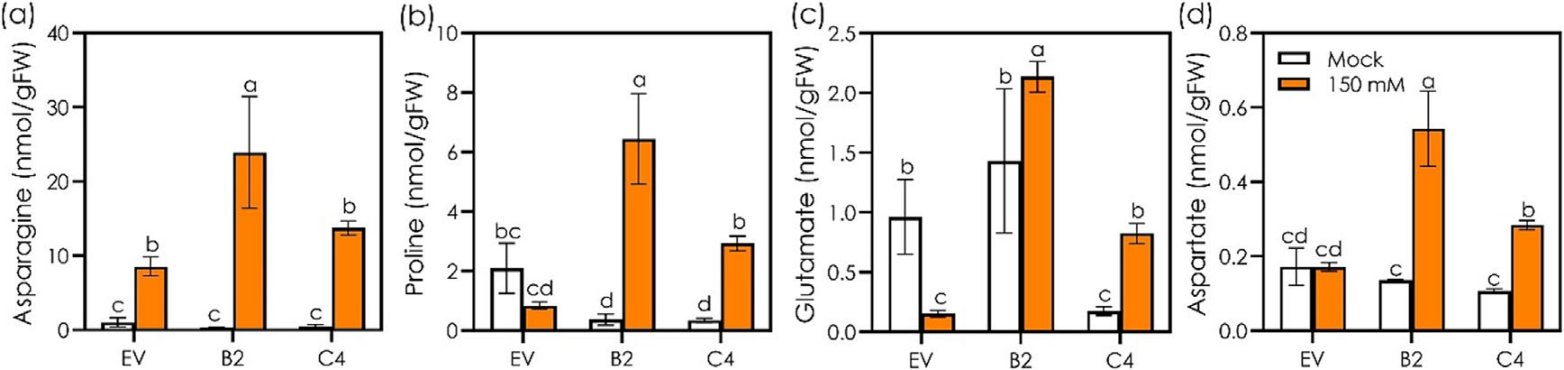
Effects of root isoprene emission on the levels of stress‐associated amino acids in the roots of non‐emitting (EV) and emitting (B2 and C4) Arabidopsis lines under control and salt‐stressed conditions. The levels of asparagine (a), proline (b), glutamate (c), and aspartate (d) were measured in root tissues of EV, B2, and C4 plants after exposure to 0 (mock) and 150 mM NaCl solutions for 4 h. all data are means ± standard deviations of 4 biological replicates. Different letters above the box plots indicate significant differences (*p* < .05) among the treatments and genotypes, based on two‐way ANOVA and Tukey's test.

The constitutive isoprene emission did not generally affect the levels of amino acids, including asparagine, glutamate, and aspartate, whereas a lower level of proline was recorded in IE lines when compared with EV line (Figure 4a–d). In the presence of severe salinity, EV line showed a significant increase in asparagine level and decline in glutamate level, while proline and aspartate levels were not statistically different in comparison with control conditions (Figure 4a–d). Salt exposure resulted in a remarkable increase in the levels of asparagine, proline, glutamate, and aspartate in B2 and C4 lines (Figure 4a–d). Organic acids, including citrate, succinate, fumarate, malate, glycerate, and glycolate, showed differential responses in both EV and IE lines under normal and salt stress conditions (Figure S5). The levels of citrate, succinate, and fumarate were significantly higher in EV line when compared with IE lines under salt‐unstressed conditions (Figure S5a–c). On the other hand, malate and glycolate levels were significantly higher in IE line B2 in comparison with EV line under control conditions (Figure S5d,e). Salt stress led to a significant decrease of citrate, succinate, and fumarate levels, while malate and glycolate levels significantly increased in EV line when compared with control conditions (Figure S5a–d, f). In IE lines, salt exposure resulted in a significant increase in citrate level in C4 line and glycerate and glycolate levels in B2 line relative to those levels found in control conditions (Figure S5a, e, f). The levels of succinate, fumarate, and malate did not show significant alteration in IE in response to salt stress when compared with control conditions (Figure S5b–d).

### 2.5 Root transcriptome profiles

**FIGURE 5:**
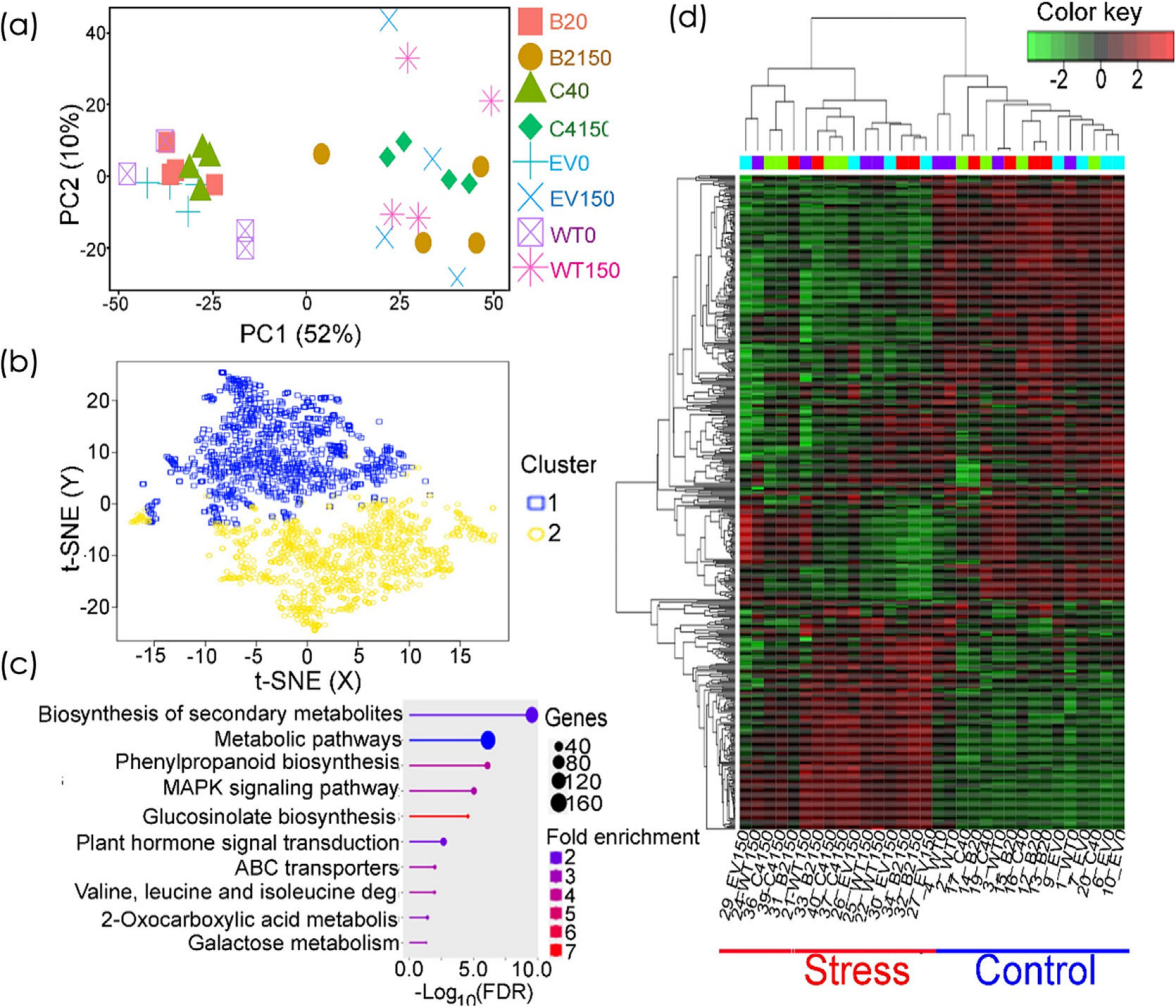
The transcriptome profiles of isoprene non‐emitter wild‐type (WT) and empty vector (EV) plants, alongside isoprene emitter transgenic lines B2 and C4 in response to 4 h treatment with 0 mM NaCl (WT0, EV0, B20, and C40, respectively) and 150 mM NaCl (WT150, EV150, B2150, and C4150, respectively). (a, b) Principal component analysis (PCA) and *t*‐distributed stochastic neighbor embedding (t‐SNE) plots of the transcriptome changes in the investigated genotypes in response to salinity stress. (c) KEGG enrichments analysis of the top 500 variable genes associated with salinity stress responses in the investigated genotypes. (d) Heatmap hierarchical clustering of the normalized expression top 100 variable genes in the investigated genotypes in response to stress and control conditions. Color bar indicates high (red) and low expression (green) levels for each genotype under different treatment conditions.

Transcriptome analysis was conducted on 10‐d‐old roots of WT and EV (NE lines) and B2 and C4 (IE lines) after a 4 h exposure to 0 mM and 150 mM NaCl. The transcriptome profiles of NE and IE genotypes were analyzed using principal component analysis (PCA) and t‐distributed stochastic neighbor embedding (t‐SNE) statistical methods to identify the transcriptomic changes induced by IE capacity, salt stress or their interaction, as depicted in Figure 5a,b. A high proportion (52%) of the variance in the transcriptome changes was made by the effect of salt and was captured by PC1, whereas a low proportion (10%) of variance was captured by PC2, representing the effect of genotypes (Figure 5a). Both PCA and t‐SNE separated the investigated genotypes exposed to 0 mM and 150 mM NaCl into main clusters (Figure 5a,b). Notably, the predominant transcriptome variations across the investigated genotypes were due to salt stress, as denoted by the larger variances in PC1 and the t‐SNE Y‐axis (Figure 5a,b). On the other hand, minor transcriptome changes were attributed to genotype‐specific effects as indicated by PC2 and t‐SNE X‐axis (Figure 5a,b).

To get in‐depth insights into the key metabolic pathway(s) interlinked with the clustering in PCA and t‐SNE for the investigated genotypes, we executed a KEGG pathway enrichment assessment of the transcriptome data using iDEP .96 (www.bioinformatics.sdstate.edu/idep96/). This KEGG enrichment revealed that the most enriched genes associated with the genotype separation in PCA and t‐SNE belonged to the “biosynthesis of secondary metabolites” and “metabolic pathways” metabolic pathways (Figure 5c). A significant number of these variable genes also aligned with metabolic pathways tied to stress response mechanisms, such as “phenylpropanoid biosynthesis,” “MAPK signaling,” “glucosinolates biosynthesis,” and the “plant hormone signal transduction pathway.” The heatmap hierarchical clustering, presented in Figure 5d, shows the top 100 variable genes across genotypes and salt stress conditions, distinctly emphasizing the pronounced impact of salinity stress.

### 2.6 Transcriptome analysis, gene ontology, KEGG enrichment, and protein–protein network

**FIGURE 6:**
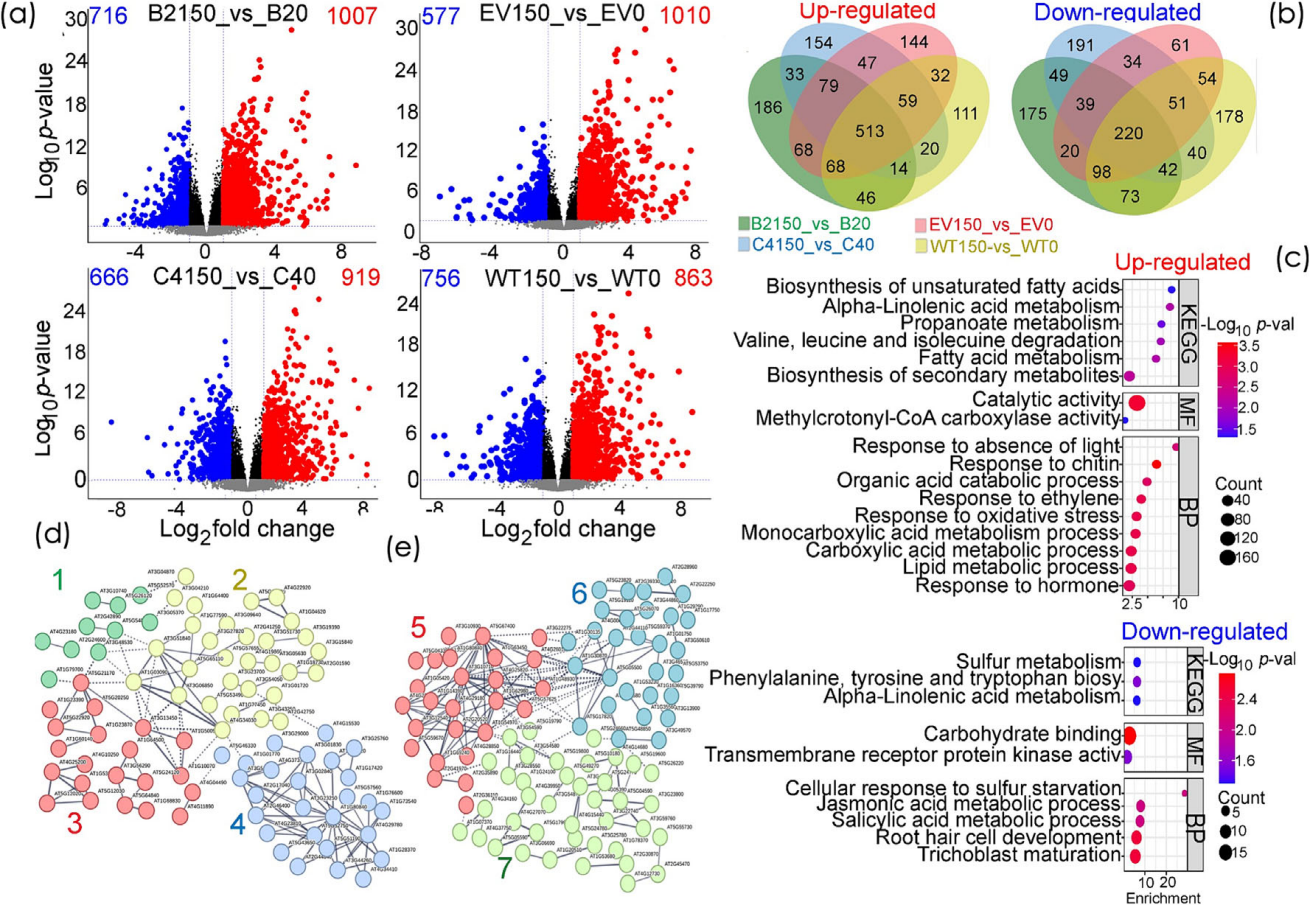
Differential expressed genes (DEGs) of isoprene non‐emitter wild‐type (WT) and empty vector (EV), and isoprene emitter transgenic lines B2 and C4 in response to 4 h treatment with 0 mM NaCl (WT0, EV0, B20, and C40, respectively) and 150 mM NaCl (WT150, EV150, B2150, and C4150, respectively). (a) Volcano plots of significantly upregulated [log_2_ (fold‐changes) ≥ 1; *q*‐values ≤.05], and downregulated [log_2_ (fold‐changes) ≤ −1; *q*‐values ≤.05] genes in “WT150 versus WT0,” “EV150 versus EV0,” “B2150 versus B20” and “C4150 versus C40” comparisons. Blue‐dashed lines represent the q‐value and fold‐change threshold. Red and blue points highlight the upregulated and downregulated genes, respectively, in the investigated comparisons. (b) Venn diagrams of overlapping DEGs in “WT150 versus WT0,” “EV150 versus EV0,” “B2150 versus B20” and “C4150 versus C40” comparisons. Four independent biological replicates (*n* = 4) were collected from each genotype under different treatments for the transcriptome analysis. (c) Gene ontology (GO) and KEGG enrichments analyses of isoprene emitter overlapping DEGs. (d, e) Protein–protein interaction networks of upregulated (d) and downregulated (e) overlapping genes in isoprene emitter transgenic B2 and C4 lines in response to salinity stress.

Log_2_ FC ≥ 1 (upregulated) and Log_2_ FC ≤ −1 (downregulated) with FDR ≤ .05 were used as minimum cutoffs to identify DEGs that are robustly regulated by isoprene emission capacity, salinity stress, or both. The DEG analysis revealed that 863 and 1010 genes were upregulated, whereas 756 and 577 genes were downregulated when comparing salt‐stressed and control NE (WT and EV, respectively) (Figure 6a). Likewise, 1007 and 919 genes were upregulated, whereas 716 and 666 genes were downregulated when comparing salt‐stressed and control IE (B2 and C4, respectively) (Figure 6a). The constructed Venn diagram illustrated that there were 733 overlapping DEGs (513 upregulated and 220 downregulated) when comparing all IE and NE genotypes under control and salt stress conditions (Figure 6b). These represent the core transcriptome changes in response to salinity stress regardless of isoprene emission capacity. In contrast, 373 (186, 33, and 154) upregulated genes and 415 (175, 49, and 191) downregulated genes were identified only in IE lines under control and salt stress conditions (B2150 vs. B2 and C4150 vs. C4). (Figure 6b). By doing this, we deliberately excluded comparisons that could introduce background or NON‐IE effects, such as EV10 versus EV and WT150 versus WT. These 788 (373 and 415) overlapping DEGs represent core specific transcriptome changes modulated by the presence of isoprene, which may contribute to change of root physiology in the presence of salinity stress (Figure 6b).

Next, we conducted gene ontology (GO) and KEGG enrichment analysis to get further insight into biological attributes of the 788 overlapping DEGs in the IE B2 and C4 lines (Figure 6c). The GO enrichment analysis using Fisher's test and Bonferroni corrections *q*‐values ≤.05 classified the 788 overlapping DEGs into two GO categories: (1) molecular functions (MF) and (2) biological processes (BP) (Figure 6c). The overlapping upregulated genes in the MF category had highly enriched GO terms related to “catalase activity”; and “methylcrotonyl‐CoA carboxylase activity,” whereas “carbohydrate binding,” and “transmembrane receptor protein kinase activity” were highly enriched GO terms among downregulated genes of the MF category (Figure 6c). Top enriched GO term (fold >2) of the upregulated overlapping DEGs in the MF category was the catalytic activity. In the BP category, highly enriched GO terms showing upregulated genes were “response to absence of light,” “response to chitin,” “response to organic acid catabolic process,” “response to ethylene,” and “response to oxidative stress” (Figure 6c). On the other hand, “cellular response to sulfur starvation,” “jasmonic acid metabolic process,” “salicylic acid metabolic process,” and “root hair cell development” were those with GO enriched terms in downregulated (Figure 6c).

With respect to KEGG enrichments, “biosynthesis of unsaturated fatty acids,” “alpha linolenic acid metabolism,” “propanoate metabolism,” “valine, leucine and isoleucine degradation” and “fatty acid metabolism,” and “biosynthesis of secondary metabolites” were among the enriched metabolic pathways in the upregulated overlapping genes (Figure 6c). On the other hand, “sulfur metabolism,” “alpha linolenic acid metabolism,” and “phenylalanine, tyrosine and tryptophan biosynthesis” were among the enriched KEGG pathways in the downregulated overlapping genes (Figure 6c). The KEGG enrichments indicated a complex regulation in the fatty acid and lipid‐related pathways in both the upregulated and downregulated genes in the IE lines, reflecting the complex nature of metabolic responses to stress conditions.

Finally, a protein–protein interaction (PPI) network analysis for the target overlapping DEGs associated with IE B2 and C4 lines in response to salinity stress was performed (Figure 6d). PPI networks demonstrated that “(1) auxin biosynthesis,” “(2) fatty acid biosynthesis and metabolism, citrate cycle, and valine, leucine and isoleucine biosynthesis and degradation,” “(3) small heat shock protein, suberin biosynthesis and B‐box zinc finger,” “(4) response to chitin, calmodulin binding protein, and ethylene‐activated signaling pathway” were clustered in the upregulated overlapping genes in IE lines (Figure 6d). Whereas PPI networks of “(5) phenylpropanoid biosynthesis and secretory peroxidase” and “(6) jasmonic acid biosynthesis process, tryptophan biosynthesis, sulfur metabolism” pathways were clustered in the downregulated overlapping genes in IE lines (Figure 6e).

## 3 DISCUSSION

While the functions of leaf‐emissions of isoprene in plant interactions with atmospheric components is well studied (Bellucci et al., 2023; Loreto & Schnitzler, 2010), the belowground role of isoprene is yet to be clarified, particularly under stress situations. Here, we demonstrate that the capacity to emit isoprene radically altered the phenotypes of the roots, stimulating root growth of IE lines under control conditions on 10‐day‐old Arabidopsis plants grown in artificial media (Figure 1d) and in 2‐week‐old Arabidopsis plants grown on soil (Figure S1). These isoprene emitting lines invested more in roots than EV NE plants (Figure 1f), showing a distinct deeper root phenotype, faster PR growth, and higher biomass (DW basis) both under control and salt‐stress conditions (Figure 1d; Figure S6). Root isoprene also appeared to affect root gravitropism in Arabidopsis (Figure S4). Our results highlight an intriguing connection: increased PR length and root biomass, together with a more rapid response to gravitropism, may indicate a more vigorous phenotype prone to rapid growth, suggesting a significant role of isoprene in modulating root morphology and environmental responsiveness.

Transgenic *N. tabacum* plants ectopically overexpressing eucalyptus *ISPS* exhibited a dwarf phenotype when compared with the EV's aboveground growth (Zuo et al., 2019). However, suppression of isoprene emission capacity in poplar resulted in slower growth and reduced apical dominance (Dani et al., 2022). It is possible that natural emitters behaved differently from *ISPS*‐overexpressing lines in resource allocation in the aboveground. Nonetheless, our results suggest that continuous isoprene emission might contribute to the shifting of resources from aboveground to belowground, resulting in a robust root growth and better salt tolerance capacity in IE lines.

There is evidence that the roots of poplar (Ghirardo et al., 2011) and *ISPS*‐overexpressing Arabidopsis (Loivamäki et al., 2007; Miloradovic van Doorn et al., 2020) emit a trace amount of isoprene. We also observed significant root isoprene emission from both IE transgenic lines compared with the EV line under control conditions (Figure 1g). *A. thaliana* IE roots showed a much lower emission than from leaves; a concentration similar to hormones (Dani et al., 2022). The non‐negligible emission of isoprene seen in EV roots may reflect non‐enzymatic isoprene formation.

Salt stress further increased the level of isoprene significantly in IE roots (Figure 1g). However, we must consider that VOC diffusion out of roots may be more restricted than from leaves where stomata are present, and that in the plates. Isoprene emission into soil cannot dissipate as quickly as isoprene emission from leaves in air. Thus, the smaller emission rate from roots might still be associated with significant effects.

It is known that isoprene emission by leaves is sustained under salt stress (Loreto & Delfine, 2000). Our results suggest a stress‐responsive role of isoprene in roots, as previously observed in the leaves, especially under heat and drought stress scenario (Brilli et al., 2007; Jardine et al., 2012; Tattini et al., 2014; Velikova & Loreto, 2005).

Isoprene synthases are located in plastids (Sharkey et al., 2013); however, recently, Zhou and Pichersky (2020) reported that a TPS‐b type *TPS47* is localized in the cytosol of tomato (*Solanum lycopersicum*). *TPS47* encoded a functional ISPS that can catalyze the formation of isoprene from DMADP in vitro. Interestingly, *TPS47* expression was identified in multiple tissues, including root tissues of tomato (Zhou & Pichersky, 2020). We infer that root isoprene emission is plausibly a common trait of the plants that harbor the *ISPS* genes in roots. However, the DMADP pool is very low in the cytosol (Weise et al., 2013), which perhaps explains why only trace amount of isoprene is emitted by roots in comparison to leaves.

Root isoprene emission in the absence of light may be different from the photosynthesis (light)‐dependent isoprene synthesis via the MEP pathway (Sharkey & Yeh, 2001). In photosynthetic organisms, photosynthesis‐independent isoprene emission at a rate similar to that emitted photosynthetically was so far identified only in microalgae growing heterotrophically on a sugar‐rich substrate (Dani et al., 2020). We showed that root isoprene emission (Figure 1g) was accompanied by a general stimulation of the levels of pyruvate and the metabolites of the MEP pathway in the root tissues of IE lines (Figure 2). This suggests that constitutive expression of *EgISPS* resulted in an increased flux of MEP pathway metabolites in the belowground organs of IE lines. Furthermore, salt‐induced enhancement of the levels of DMADP and IDP corresponded with an increased emission of isoprene from the roots of both transgenic lines. Many of the MEP pathway enzymes are light‐dependent (Sharkey & Yeh, 2001) and isoprene emission from leaves has even been modeled based on light‐dependent generation of reducing power (Morfopoulos et al., 2014). Thus, the capacity of roots to emit isoprene may be based on a specific pathway whose regulation is different from that of the leaves. Indeed, our KEGG pathway analysis also revealed an upregulation of DXS in IE roots under salt stress (Figure S7a), suggesting a MEP pathway activation at the transcript level in the roots. DXS catalyzes the first committed step of MEP pathway, which is known to be crucial in maintaining a continuous flux of MEP pathway metabolites (Banerjee & Sharkey, 2014).

The higher PR length was correlated with the enhanced biosynthesis of growth hormones in the roots of IE lines. In comparison with the NE EV, IE lines maintained a consistently high level of CKs (tZT, tZTR, and iPR) in their roots under both normal and saline conditions. Dani et al. (2022) also reported an increased level of CKs (iP and iPR) in the leaves of IE poplars compared with transgenic NE plants. KEGG analysis revealed an upregulation of zeatin synthesis in IE lines under control condition (Figure S8a). We propose that isoprene‐induced high level of CKs has significant effect on faster growth of PR, and thus plays beneficial roles in short‐term salinity tolerance. We also observed an enhanced level of IAA in the roots of IE transgenic lines under salt stress conditions. These results suggest that isoprene might have interfered with the CK‐auxin crosstalk in maintaining root meristem size, ensuring root growth (Moubayidin et al., 2009), and controlling root development (Dello Ioio et al., 2008).

Interestingly, the levels of JA‐associated metabolites like OPDA, JA, and JA‐Ile and SA conjugate (SAG) were significantly decreased while the ABA level markedly increased in both IE lines upon exposure to salt stress. KEGG analyses showed an upregulation of several components of JA synthesis in unstressed IE roots (Figure S8b), and this supported the high JA levels detected in unstressed IE roots. High levels of JA‐related metabolites (OPDA, JA, JA‐Ile) in unstressed IE roots, suggest a defense priming effect of isoprene as also demonstrated in leaves (Monson et al., 2021; Zuo et al., 2019). Thus, isoprene might have stimulated a general activation of stress hormones (e.g., ABA) but a lower activation of induced stress responses involving systemic induced resistance (by JA) or systemic acquired resistance (by SA) (Saxena et al., 2020).

Metabolomic analysis also revealed salt‐induced upregulation of the stress metabolites like proline, aspartate, and glutamate, as well as the amino acid asparagine. Amino acids, including proline, aspartate, and glutamate are well‐known salt‐tolerant metabolites, acting as osmolytes to support plants under salt‐induced osmotic stress (Rai, 2002). Additionally, KEGG analysis showed an upregulation of plant hormone signal transduction pathways mediated by zeatin, ABA, auxin, and JA in the IE roots (Figure S7b), which might be crucial for persistent growth of PR when exposed to salt stress (Figure 1a).

Acquisition of the capacity to emit isoprene may alter gene expression aboveground (Behnke et al., 2010), and the possibility that isoprene acts directly as a signaling molecule was proposed by Harvey and Sharkey (2016). Previous transcriptomic analysis of unstressed isoprene‐fumigated Arabidopsis leaves showed that isoprene acts as a signaling molecule upstream of many growth regulators involved in various signaling cascades (Zuo et al., 2019). In isoprene‐treated Arabidopsis leaves, there was an upregulation of JA signaling/biosynthesis genes (Zuo et al., 2019), whereas in IE roots, we observed a downregulation of *ROXY20*, which negatively regulates JA signaling and a wounding‐inducible JA‐related gene (*CYP84A4*) (Table S4). Moreover, we observed an upregulation of ABA‐associated genes (*CYP709B2*, *ARCK1*, and *BBD2*). Interestingly, isoprene‐fumigated Arabidopsis leaves showed a downregulation of several ABA‐related genes (Zuo et al., 2019). For instance, the *bifunctional nuclease in basal defense response 2 (BBD2)*, which is involved in JA and ABA signaling for drought tolerance (Huque et al., 2021), was downregulated (Zuo et al., 2019) while it was upregulated in salt‐stressed roots of IE lines (Table S4). Root IE capacity upregulated the expression of genes associated with gibberellin deactivation (*ATGA2OX1*), brassinosteroid signaling (*JMJD5*), but downregulated *methyl esterase 1* (*MES1*), which is known to encode MES1 that plays a role in methylsalycylate (MeSA) or methyl indole‐3‐acetic acid (Me‐IAA) hydrolysis (Vlot et al., 2008; Yang et al., 2008) (Table S5). Exogenous Me‐IAA (the inactive form of IAA) was found to significantly inhibit root growth in plant (Yang et al., 2008).

In the IE B2 and C4 lines, we found an enhanced expression of several key genes associated with the defense against biotic and abiotic stresses, as also reported by Zuo et al. (2019) in isoprene‐treated Arabidopsis leaves. Particularly, we observed an alteration of associated genes involved in general defense responses (*G1IP‐LIKE*, *PTF1*, *SPA2*, *ROXY20*, and *NF‐YC13*), in biotic stress responses (*LOV1*, *MAGL6*, and *CYP709B2*) and in abiotic stress response, like salt stress (*ARCK1* and *CYP709B2*), light (*HYR1* and *OEP6*), temperature (*JMJD5*), oxidative (*MDAR4* and *ELT3*), osmotic (*ARCK1*, *CYP709B2*, *MAGL6*, *NAC080*, and *CYP84A4*), and drought (*BBD2*) (Table S4). Finally, protein networks analysis revealed an upregulation of small heat shock protein and calmodulin binding proteins in IE lines, which perhaps contributed to enhanced salt tolerance of IE lines.

## 4 CONCLUSION

A tiny emission of isoprene from roots can be measured; given reduced diffusion through soil this could have physiological significance. A high investment of biomass into roots was observed as a consequence of constitutive root isoprene emission. Possible mechanisms include (i) the enhancement of the levels of hormones, especially CK; (ii) improved synthesis of stress tolerance metabolites like proline, aspartate, and glutamate, and (iii) modulation of the hormone signaling pathways (e.g., JA, SA, CKs, and ABA) and associated gene expression. These findings suggest the onset of pleiotropic effects when isoprene is emitted, affecting gene expression, hormone synthesis, and the phenotypes of plants. In particular, the belowground signaling role of isoprene modulating hormones and protecting roots from stresses opens up a promising avenue for future research on isoprene. In accordance with Sharkey and Monson (2017), our findings also imply that isoprene has a general role in regulating biological processes that are critical for conferring plant stress resilience. Further comparative studies of transgenic lines that are capable or defective in isoprene emission across diverse plant species will provide deeper insights into the specific mechanisms by which isoprene alters root physiology to improves plant resistance to salinity and other soil‐borne stresses.

## 5 MATERIALS AND METHODS

### 5.1 Plant materials, growth conditions, and salt treatments

We used our previously developed *A. thaliana* *ISPS*‐transgenic lines (Zuo et al., 2019). The full complementary *ISPS* DNA sequence including native transit peptide sequence from *Eucalyptus globulus* was placed under the *Arabidopsis* rubisco small subunit promoter rbcS‐1A (*At1g67090*) to generate the transgenic, IE lines. After the *ISPS* sequence, an *octopine synthase* gene was placed as a transcriptional terminator of the transgene. The construct without *EgISPS* was also transformed with the vector to generate a NE EV control. After successful transformation, seven transgenic lines and three empty vector lines were characterized. Among the transgenic lines, B2 and C4 showed highest level of isoprene emission (Zuo et al., 2019). Thus, we selected B2 and C4 transgenic lines as IE, together with EV‐B3 (referred as EV hereafter) as NE for the current study.

The *A. thaliana* seeds were surface sterilized with 70% ethanol and 5% bleach solution for 5 and 10 min, respectively, followed by washing with sterilized milli‐Q water five times. Seeds of EV and IE (B2 and C4) lines were placed on germination medium (GM) plates containing ½ Murashige and Skoog (MS) solid medium (.8% agar and 1% sucrose), supplemented with Gamborg's vitamins solution (Sigma‐Aldrich, Germany). Each plate contained 12 seeds, and four plates were used for each genotype to collect one set of data for each parameter. After stratification for 3 days at 4°C in the dark, the plates were vertically placed in the growth chamber under a 16 h: 8 h, light: dark photoperiod, 120 μmol m^−2^ s^−1^ photosynthetically active radiation (PAR), 23/20°C day/night temperatures, and 60% relative humidity. To simulate salt stress, EV, B2, and C4 seedlings were exposed to different concentrations of sodium chloride (NaCl) based on the type of investigation. For root phenotype analyses and isoprene emission measurements, five‐day‐old seedlings were treated with 0 (mock), 50 (mild stress), or 100 (severe stress) mM NaCl for 5 days in solid MS medium.

To study the primary root growth in soil rather than in an artificial media, NE (EV) and IE (B2 and C4) Arabidopsis plants were planted in homemade Rhizoboxes, special boxes where root growth can be monitored and photographed. Plants were germinated and grown for 2 weeks in sterile Suremix soil (Michigan Grower Products Inc, Galesburg, MI, USA) and kept in the same controlled growth chambers used for plate experiments.

For determining root gravitropic response, five‐day‐old NE (EV), and IE (B2 and C4) plants were transferred to new GM plates with fresh MS medium and grown for one additional day. Plates were rotated at 90° immediately after transferring the plants, and the photographs of the roots were recorded at different time points (0, 2, 5, 7, and 24 h) for analyzing the root angles.

For RNA‐seq, metabolite, and hormone analyses, NE (EV) and IE (B2 and C4) seedlings were grown for 10 days as described for phenotype analysis. The roots of the seedlings were then soaked in 0 or 150 mM NaCl solutions for 4 h. For RNA‐seq analysis, the NE WT was additionally tested, in the same conditions of the other investigated genotypes. This test was intended to verify that the EV did not cause any reprogramming of the transcriptome, confirming previous findings (Dani et al., 2022; Zuo et al., 2019).

### 5.2 Measurement of isoprene emission from roots

After 5 days of salt stress treatments, roots were harvested, and 50 mg of the samples were collected in 5 mL glass vials. Roots were incubated for 4 h at 32°C and a PAR of 300 μmol m^−2^ s^−1^, as described in Miloradovic van Doorn et al. (2020). Isoprene emission was measured in the headspace of the vial with the Fast Isoprene Sensor (FIS, Hills Scientific, Boulder, CO, USA). The headspace gas (3 mL) was injected into air flowing into the instrument at 400 mL min^−1^ by a gastight syringe. The FIS calibration was carried out using a 3.225 ppm isoprene standard (Airgas USA LLC, TX, USA). Isoprene emission was calculated per gram of root fresh weight. Three independent experiments were carried out to validate the data of isoprene emission from the roots.

### 5.3 Metabolites estimation and analysis

#### 5.3.1 MEP pathway metabolites

The harvested roots were ground using a tissue‐lyser followed by an extraction with an extraction buffer containing acetonitrile: isopropanol: 20 mM ammonium bicarbonate (3:1:1). After centrifugation at 14,000 *g* for 10 min, the supernatants were collected for analyzing MEP pathway metabolites. A volume of 200 μL supernatant was transferred to glass inserts placed in 2 mL glass vials for LC–MS/MS analysis. MEP pathway metabolites, including 1‐deoxy‐d‐xylulose 5‐phosphate (DXP,) 2‐C‐methyl‐d‐erythritol 4‐phosphate (MEP), methylerythritol cytidyl diphosphate (CDP‐ME), 2‐C‐methyl‐d‐erythritol‐2,4‐cyclodiphosphate (MEcDP), 4‐hydroxy‐3‐methyl‐butenyl 1‐diphosphate (HMBDP), isopentenyl diphosphate (IDP), and dimethylallyl diphosphate (DMADP) were quantified by an Acquity TQD Tandem Quadrupole Mass Spectrometer with an Agilent InfinityLab Poroshell 120 HILIC‐Z, column (2.1 × 100 mm, 2.7 μ, Agilent, Santa Clara, CA, USA) following the protocol described by Sahu et al. (2023). Commercial DXP, MEP, CDP‐ME, MEcDP, HMBDP, IDP, and DMADP (Logan, UT, USA) were used to develop response curves for calculating the levels of MEP pathway metabolites. The levels of the MEP pathway metabolites were calculated based on FW.

#### 5.3.2 Amino acids and organic acids analyses

The extraction of root samples was carried out according to the protocol described by Xu et al. (2021, 2022) with slight modifications. Frozen root samples were ground into a fine powder using a tissue‐lyser. The extraction was carried out in chloroform:methanol (3:7) solution for 2 h at −20°C with vortexing every 30 min. D‐[UL‐^13^C_6_) fructose 1, 6‐bisphosphate and norvaline were added to the sample tubes as internal standards. A volume of 300 μL ice‐cold water was added to each tube for extraction of water‐soluble metabolites. The Eppendorf tubes were vortexed for 20 s followed by centrifugation at 4200 *g* for 10 min. The upper methanol–water phase was separately aliquoted into Eppendorf tubes followed by lyophilization to dryness and stored at −80°C for GC–MS analysis.

For determining the levels of pyruvate, amino acids, and organic acids by GC–MS, we initiated the process by derivatizing the samples with the addition of methoxyamine hydrochloride dissolved in dry pyridine. The mixture was kept at 60°C for 15 min, then cooled for 10 min. Subsequently, it was subjected to silylation by introducing *N*‐tert‐bulydimethylasyl‐*N*‐methyl‐trifluoracetamid with 1% (w/v) tert‐bultylmethylchlorisilane, and kept at 60°C overnight, resulting in trimethylsilyl (TBDMS) derivatives. The derivatized samples were then subjected to analysis by an Agilent 7890 GC system (Agilent, Santa Clara, CA, USA) coupled to an Agilent 5975C inert XL Mass Selective Detector (Agilent, Santa Clara, CA, USA) with an autosampler (CTC PAL; Agilent, Santa Clara, CA, USA) following a published protocol (Xu et al., 2022). The characteristic fragment ions used for measuring the metabolites are detailed in Table S1. We have quantified the levels of amino acids and organic acids based on FW.

#### 5.3.3 Hormone contents

Root samples were homogenized using a tissue‐lyser, and then extracted in a buffer containing 4:1 methanol: MilliQ water (v/v) added with butylated hydroxytoluene and formic acid. Samples were quantified by Acquity TQD Tandem Quadrupole Mass Spectrometer with an Acquity BEH Amide column (1.7 μm × 2.1 mm × 50 mm) (Acquity Group, Waters, Milford, MA, USA) with an autosampler (2777C, Waters, MA, USA). Salicylic acid (SA), 12‐hydroxy jasmonic acid (12OH‐JA), jasmonic acid d‐5 (JA d‐5), abscisic acid d‐6 (ABA d‐6), 12‐hydroxy‐jasmonoyl‐isoleucine (12OH‐JA‐Ile), and ^13^C6‐indole‐3‐acetic acid (IAA‐^13^C6) were used for developing the standard curves. For the gradient sequence of most of the hormones (excluding cytokinins) see Table S2. For quantification of CKs, namely trans‐zeatin (tZ), trans‐zeatin‐riboside (tZR), and isopentenyl‐riboside (iPR), root tissues were ground and extracted by 8:2 methanol: MilliQ water (v/v). After centrifugation at 13,000 *g* for 5 min, the supernatants were collected and evaporated in a Savant SpeedVac (Thermo‐Fisher Scientific, Waltham, MA, USA) at maximum speed for 2 h. Dried extracts were resuspended in 10% acetonitrile. The samples were run by an Acquity Xevo TQ‐XS UPLC/MS/MS (Waters, Milford, MA, USA) and separated with a BEH C18 2.1 × 50 mm column. Caffeine ^13^C_3_ was used as standard. Water and .1% formic acid (A) and acetonitrile (B) were used as mobile phase. For cytokinins a different gradient was used, see Table S3. The mass‐spectra acquisition setup included positive mode electrospray ionization mode (ES+), source temperature of 150°C, and desolvation temperature of 400°C. Collision gas and nebulizer gas flow were set to .17 mL min^−1^ and 7 bar, respectively. Gas flow for the desolvation and cone was set to 800 and 150 L h^−1^, respectively. Scan time was 100 to 200 amu s^−1^. Finally, the levels of examined hormones were calculated and expressed on FW basis.

#### 5.3.4 LC–MS/MS and GC–MS/MS data analysis

The MassLynx 4.0 and GC/MSD Chemstation (Agilent, Santa Clara, CA, USA) were used to acquire the LC–MS/MS and GC–MS data, respectively. The specific metabolites were identified by their mass to charge (m/z) ratio and retention time using authentic standards. Standard curves were developed using the authentic standards available for each targeted metabolite. Both LC–MS and GC–MS datasets were converted to the MassLynx format, and QuanLynx software was then used to analyze the data, including peak detection and quantification of the metabolites. Absolute quantification of the metabolites was carried out using external standard curves that were normalized with the internal standards.

### 5.4 Plant phenotype and growth analyses

Plant growth parameters and dry weight (DW) of both roots and shoots were determined after 5 days of exposure to NaCl solutions. For measuring DW, roots and shoots were separated using a razor blade and transferred to paper bags. The paper bags were oven dried at 65°C for 3 days and the DW of roots and shoots was then recorded using a digital balance. Root/shoot ratio and percent reduction of root biomass were calculated based on DW. Root phenotype parameters, including PR length, LR number, and root growth were measured by analyzing the photographs taken after 5 days of stress treatments. For the phenotypic parameters all the plants in the plate were evaluated, harvested, and analyzed for a total of 48 individual plants. For each parameter, three independent experiments were carried out under the same experimental conditions. All three independent replicates showed similar results, and the data from one representative experiment were presented in the graph (48 plants). For Rhizobox analyses, 2‐week‐old plants were explanted, and pictures were taken for PR length measurements. All root phenotype (plates and soil) parameters were measured using *Fiji* software (Schindelin et al., 2012). All measurements were replicated at least three times.

### 5.5 Gene expression analyses

#### 5.5.1 RNA extraction

Total RNA from frozen root samples was extracted using the RNeasy Mini Kit (Qiagen, Hilden, Germany). The RNA concentration, integrity, and quality were determined with a Qubit RNA Broad‐Range Assay Kit (Invitrogen, Waltham, MA, USA) and a Qubit 4 benchtop fluorometer (Invitrogen, Waltham, MA, USA). RNA integrity was further assessed with a 2100 Bioanalyzer (Agilent Technologies, Santa Clara, CA, USA). All samples used for sequencing had an RNA integrity number of at least 8.5 (1–10; low to high quality).

#### 5.5.2 Library preparation and RNA‐sequencing analysis

mRNA sequencing was performed at the Michigan State University Research Technology Support Facility Genomics Core (https://rtsf. natsci.msu.edu/genomics/). Libraries were prepared using the Illumina Stranded mRNA Library Preparation, Ligation Kit with IDT for Illumina Unique Dual Index adapters following the manufacturer's recommendations except that half volume reactions were used. Completed libraries were quality checked and quantified using a combination of Qubit dsDNA high sensitivity (HS) and Agilent 4200 Tape Station HS DNA1000 assays. The libraries were pooled in equimolar proportions and the pool quantified using the Invitrogen Collibri Quantification qPCR kit. The pool was combined with other pools of Illumina Stranded RNA libraries prepared by the Genomics Core to make use of a shared S4 lane. This combined pool was likewise quantified using the Invitrogen Collibri Quantification qPCR kit. The combined pool was loaded onto one lane of an Illumina S4 flow cell and sequencing was performed in a 2 × 150 bp paired end format using a NovaSeq 6000 v1.5, 300 cycle reagent kit. Base calling was done by Illumina Real Time Analysis (RTA) v3.4.4 and output of RTA was demultiplexed and converted to FastQ format with Illumina Bcl2fastq v2.20.0. The paired‐end raw sequences obtained from RNA‐seq of WT, EV, B2, and C4 grown under both normal (0 mM NaCl) and salt‐stress (150 mM NaCl) conditions were checked using FastQC v0.12.1, then trimmed using fastp (Chen et al., 2018) to remove sequences with Q scores <30. High quality reads were aligned to Athaliana_447_TAIR10.fa reference genome (phytozome‐next.jgi.doe.gov/) using STAR 2.7.11a (Dobin et al., 2013). The STAR‐output mapped reads were then subjected to featureCounts (Liao et al., 2014), a read summarization program to counts the number of reads mapping to each genomic feature. Estimate variance–mean dependence in read count data and test for differential expressed genes (DEGs) based on negative binomial distribution model was performed using DESeq2 (Love et al., 2014). False discovery rate (FDR < .05) and fold change (log_2_ FC ≥ 1.0 or log_2_FC ≤ −1) were used as the minimum cutoffs to determine DEGs among different comparisons. For normalization of the read counts, we applied a variance stabilizing transformation (VST). This approach was used to stabilize the variance across our dataset. Subsequently, the 6000 most variable genes were identified and selected for further analysis. These genes were chosen due to their high variability among the different genotypes, indicating their potential biological significance. We utilized this subset of genes for constructing heatmap clustering, principal component analysis (PCA), and t‐SNE to explore and visualize the data structure and relationships using iDEP .96 bioinformatic tool (http://bioinformatics.sdstate.edu/idep96/).

### 5.6 Statistical analyses

To statistically determine the effect of isoprene on phenotype (PR length, LR number, FW, DW, and root/shoot ratio), and on the levels of hormones, amino acids, organic acids, and MEP metabolites, data were analyzed by two‐way analysis of variance (ANOVA) with post hoc Tukey's test. PR length reduction, DW reduction of roots, and root bending/curvature were analyzed by two‐way ANOVA with Tukey's test. All differences among means were considered statistically significant at *p* < .05. Volcano plot analysis was carried out using “EnhancedVolcano” package in RStudio v. 2023.09.0. Protein–protein interaction networks were generated by using multiple proteins search function and *Arabidopsis* as model organism in string database, accessible at (https://string-db.org/).

## AUTHOR CONTRIBUTIONS

MB, MGM, and TDS conceived the study and designed the experiments. SMW executed preliminary studies. MB, MGM, and YZ carried out experimental works. MB, MGM, and MA analyzed the data. MB and MGM wrote the original manuscript that was then revised by FL and TDS. TDS, FL, and LDG provided chemicals, reagents, and other research support. All authors revised and approved the manuscript.

## CONFLICT OF INTEREST STATEMENT

The Authors did not report any conflict of interest.

## CONFLICT OF INTEREST STATEMENT

No potential conflict of interest was declared.

## Supporting information


**Data S1.** Peer Review.

**Data S2.** Supporting Information.

**Table S1.** Parameters for transitions of measured metabolites in LC–MS/MS and GC–MS. Multiple reaction monitoring (MRM) is used for Ion‐pair chromatography–tandem mass spectrometry (IPC‐MS/MS), with a dwell time of 20 ms set for each transition. Q1, m/z of the precursor ion; Q3, m/z of the product ion. Cone and collision energy were optimized by direct infusion of standards. Selected ion monitoring (SIM) is for GC–MS. *Pyruvate was measured by GC–MS based on tert‐butyldimethylsilyl (TBDMS) derivatization.
**Table S2.** For OPDA, JA, JA‐ile, MeJA, ABA, IAA, SA, SAG determination.
**HPLC column:** Ascentis Express C18 2.1x50 mm (2.7 μm particle size) [Sigma cat# 53822‐U]. Use with .5 μm precolumn filter (IDEX Health and Science A‐318 (filter holder), A‐102 (frit), U‐288 (male‐to‐male coupler). This column has a pressure limit of 6000 psi (413 bar).
**Table S3.** For tZ,TZR,iPR determination.
**Table S4.** Supporting Information.
**Table S5.** Supporting Information.

## Data Availability

All the data are available in the main text and in the Supporting Information. All data related to RNA sequencing is available at https://www.ncbi.nlm.nih.gov/geo/query/acc.cgi?acc=GSE270516.
