# Supplementary material for: The effect of constitutive root isoprene emission on root phenotype and physiology under control and salt stress conditions
Source: Plant Direct. 2024 Jul 6;8(7):e617. doi: 10.1002/pld3.617 (PMC11227114; doi:10.1002/pld3.617)
Supplement: Supplementary file 3 — Table S1. Parameters for transitions of measured metabolites in LC–MS/MS and GC–MS. Multiple reaction monitoring (MRM) is used for Ion‐pair chromatography–tandem mass spectrometry (IPC‐MS/MS), with a dwell time of 20 ms set for each transition. Q1, m/z of the precursor ion; Q3, m/z of the product ion. Cone and collision energy were optimized by direct infusion of standards. Selected ion monitoring (SIM) is for GC–MS. *Pyruvate was measured by GC–MS based on tert‐butyldimethylsilyl (TBDMS) derivatization. Table S2. For OPDA, JA, JA‐ile, MeJA, ABA, IAA, SA, SAG determination. HPLC column: Ascentis Express C18 2.1x50 mm (2.7 μm particle size) [Sigma cat# 53822‐U]. Use with .5 μm precolumn filter (IDEX Health and Science A‐318 (filter holder), A‐102 (frit), U‐288 (male‐to‐male coupler). This column has a pressure limit of 6000 psi (413 bar). Table S3. For tZ,TZR,iPR determination. Table S4. Supporting Information. Table S5. Supporting Information. [file PLD3-8-e617-s003.docx]

**Bellucci M., Mostofa M.G, Weraduwage S.M, Xu Y, Abdelrahman M, De Gara L, Loreto F, *Sharkey TD (2023) The effect of constitutive root isoprene emission on root phenotype and physiology under control and salt stress conditions**

*Corresponding author: Thomas D. Sharkey ([tsharkey@msu.edu](mailto:tsharkey@msu.edu))

**Supplementary Tables**

**Table S1**

| **Multiple reaction monitoring (MRM) with LC-MS/MS** | | | |
| --- | --- | --- | --- |
| **Metabolites** | **Q_1_[Q_3_] (m/z) of mass isotopomers** |  |  |
| **DXP** | 213[79] |  |  |
| **MEP** | 215[79] |  |  |
| **CDP-ME** | 520[322] |  |  |
| **MEcDP** | 277[79] |  |  |
| **HMBDP** | 261[79] |  |  |
| **IDP+DMADP** | 245[79] |  |  |
| **OPDA** | 291[165] |  |  |
| **JA** | 209[59] |  |  |
| **JA-Ile** | 322[130] |  |  |
| **MeJA** | 225[151] |  |  |
| **ABA** | 263[153] |  |  |
| **IAA** | 176[130] |  |  |
| **TZTR** | 352[220] |  |  |
| **IPR** | 336[204] |  |  |
| **TZT** | 220 [136] |  |  |
| **SAG** | 299[137] |  |  |
| **SA** | 137[73] |  |  |
| **ABA-d6** | 269[159] |  |  |
| **JA-d5** | 214[62] |  |  |
| **^13^C_6_-SA** | 143[99] |  |  |
| **Selected ion monitoring (SIM) with GC-MS** | |  |  |
| **Metabolite** | **Mass range** |  |  |
| **Pyruvate*** | *174* |  |  |
| **Asparagine** | *417* |  |  |
| **Proline** | *286* |  |  |
| **Glutamate** | *432* |  |  |
| **Aspartate** | *302* |  |  |

**Table S1**

Parameters for transitions of measured metabolites in LC-MS/MS and GC-MS. Multiple reaction monitoring (MRM) is used for Ion-pair chromatography–tandem mass spectrometry (IPC-MS/MS), with a dwell time of 20 ms set for each transition. Q1, m/z of the precursor ion; Q3, m/z of the product ion. Cone and collision energy were optimized by direct infusion of standards. Selected ion monitoring (SIM) is for GC-MS. *Pyruvate was measured by GC-MS based on tert-butyldimethylsilyl (TBDMS) derivatization.

**Table S2**

**For OPDA, JA, JA-ile, MeJA, ABA, IAA, SA, SAG determination**

**HPLC column**: Ascentis Express C18 2.1x50 mm (2.7 μm particle size) [Sigma cat# 53822-U]. Use with 0.5 μm precolumn filter [IDEX Health and Science A-318 (filter holder), A-102 (frit), U-288 (male-to-male coupler). This column has a pressure limit of 6000 psi (413 bar).

**Column Temperature**: 40°C

**Mobile phase solvents**: A) Water + 0.1% formic acid; B) Methanol

**LC gradient**

| **Time (min)** | **Flow rate (mL/min)** | **% A** | **%B** |
| --- | --- | --- | --- |
| 0 | 0.400 | 98 | 2 |
| 0.5 | 0.400 | 98 | 2 |
| 3.0 | 0.400 | 30 | 70 |
| 3.5 | 0.400 | 0 | 100 |
| 4.0 | 0.400 | 0 | 100 |
| 4.01 | 0.400 | 98 | 2 |
| 5.0 | 0.400 | 98 | 2 |

**Table S3**

**For tZ,TZR,iPR determination**

**HPLC column:** Waters Acquity BEH C18 2.1 x 50 mm column. High pressure limit: 15000.0 psi

**Column Temperature**: 40°C

**Mobile phase solvents**: A) Water + 0.1% formic acid; B) Acetonitrile

| **Time (min)** | **Flow rate (mL/min)** | **% A** | **%B** |
| --- | --- | --- | --- |
| 0 | 0.400 | 98 | 2 |
| 0.5 | 0.400 | 98 | 2 |
| 4.0 | 0.400 | 60 | 40 |
| 5.0 | 0.400 | 30 | 70 |
| 6.0 | 0.400 | 1 | 99 |
| 7.0 | 0.400 | 1 | 99 |
| 7.01 | 0.400 | 98 | 2 |
| 8.0 | 0.400 | 98 | 2 |

**Table S4**

| **Gene altered by isoprene** | | | | | |  |
| --- | --- | --- | --- | --- | --- | --- |
| General | Fungus and bacterium | Salt | Heat | Oxidative | Osmotic | |
| ***PTF1*^1^**  ***SPA2*^2^**  **A*RCK1*^3^**  *CYP84A4*^4^  *DUF241*^5^  *ROXY20*^6^  *NF-YC13*^7^ | **LOV1^8^**  **MAGL6^9^**  *ELT3*^10^  **SPA2^2^**  **ARCK1^3,16^** | ***ARCK1*^3^**  ***CYP709B2*^22^** | ***JMJD5*^12,18^** | ***OEP6*^11^**  ***MDAR4*^13^**  *ELT3*^10^ | ***ARCK1*^3^**  ***MAGL6*^9,17^**  ***NAC080***^14^  *CYP84A4*^4^ | |

Genes that were differentially expressed B2 and C4 roots compared with WT and EV, are tabulated based on the roles of these genes in general defense response and biotic and abiotic stress responses. Bold and regular fonts denote isoprene-mediated up-and down-regulated genes, respectively. For RNA-sequencing, roots were harvested from 10-d-old *Arabidopsis* seedlings exposed to 0 mM (unstressed) and 150 mM NaCl (salinity stress) for four h (*n*= 4 per genotype). Numbers in superscript denote the corresponding references. *G1P-LIKE*, *get1-interacting protein-LIKE*; *PTF1*, *plastid transcription factor 1*; *SPA2*, *SPA1-related 2*; ARK1; *ABA and osmotic-stress-inducible receptor-like cytosolic kinase 1*, *CYP84A4* and *CYP709B2* *cytochrome p450 superfamily*; *DUF241*, *domain of unknown function 241*; ROXY20, *CC-type glutaredoxin*; *NF-YC13*, *nuclear factor Y*; LOV1, *locus orchestrating victorin effects1*, MAGL6, *monoacylglycerol lipase 6*; *ELT3,* *esterase/lipase/thioesterase 3*; HYR1, *hypostatin resistance 1*; OEP6, *outer envelope protein 6*, *JMJD5*, *jumonji c domain-containing protein 3*; *MDAR4*, *monodehydroascorbate reductase 4*; *NAC080*, *nac domain containing protein 80*.

**Table S5**

| **Gene altered by isoprene** | | | | | |
| --- | --- | --- | --- | --- | --- |
| JA | ABA | Gibberellin | Brassinosteroids | MeSA | MeIAA |
| C*YP84A4*^4^  *ROXY20*^6^ | ***CYP709B2*^11,25^**  ***ARCK1*^3^**  ***BBD2*^21^** | **A*TGA2OX1*^16^** | **JMJD5^12,18^** | *MES1*^19,20^ | *MES1*^19,20^ |

Genes that were differentially expressed in B2 and C4 roots compared with WT and EV, are tabulated based on the roles of these genes in signaling and synthesis of growth regulators. Bold and regular fonts denote isoprene-mediated up-and down-regulated genes, respectively. For RNA-sequencing, roots were harvested from 10-d-old Arabidopsis seedlings exposed to 0 mM (unstressed) and 150 mM NaCl (salinity stress) for four h (n= 4 per genotype). Numbers in superscript denote the corresponding references. A*TGA2OX1I*, *Arabidopsis thaliana gibberellin 2-oxidase 1*; *MES1*, *methyl esterase I*.

**References**

1. **Hur YS, Kim J, Kim S, Son O, Kim WY, Kim GT, Ohme-Takagi M, Cheon CI. 2019.** Identification of TCP13 as an upstream regulator of *ATHB12* during leaf development. *Genes*, **10**: 644.
2. **Chen S, Wirthmueller L, Stauber J, Lory, N, Holtkotte X, Leson L, Schenkel C, Ahmad M, Hoecker U. 2016.** The functional divergence between SPA1 and SPA2 in Arabidopsis photomorphogenesis maps primarily to the respective N-terminal kinase-like domain. *BMC Plant Biology*, **16**: 165.
3. **Tanaka H, Osakabe Y, Katsura S, Mizuno S, Maruyama K, Kusakabe K, Mizo J, Shinozaki K, Yamaguchi-Shinozaki K. 2012**. Abiotic stress-inducible receptor-like kinases negatively control ABA signaling in Arabidopsis. *The Plant Journal*, **70**: 599–613.
4. **Weng JK, Li Y, Mo H, Chapple C. 2012**. Assembly of an evolutionarily new pathway for α-pyrone biosynthesis in Arabidopsis. *Science,* **337:** 960–964.
5. **Savage LJ, Imre KM, Hall DA, Last RL. 2013.** Analysis of essential Arabidopsis nuclear genes encoding plastid-targeted proteins. *PLoS ONE* **8**: e73291.
6. **Zander M, Chen S, Imkampe J, Thurow C, Gatz C. 2012.** Repression of the Arabidopsis thaliana jasmonic acid/ethylene-induced defense pathway by TGA-interacting glutaredoxins depends on their C-terminal ALWL motif. *Molecular Plant*, ***5***: 831–840.
7. **Riechmann JL, Heard J, Martin G, Reuber L, Jiang C, Keddie J, Adam L, Pineda, O, Ratcliffe OJ, Samaha RR, Creelman R, Pilgrim M, Broun P, Zhang JZ, Ghandehari D, Sherman BK, Yu G. 2000.** Arabidopsis transcription factors: genome-wide comparative analysis among eukaryotes. *Science,* **290**, 2105–2110.
8. **Sweat TA, Lorang JM, Bakker EG, Wolpert, TJ**. **2008.** Characterization of natural and induced variation in the LOV1 gene, a CC-NB-LRR gene conferring victorin sensitivity and disease susceptibility in Arabidopsis. *Molecular Plant-Microbe Interactions,* **21**: 7–19.
9. **Kim RJ, Kim HJ, Shim D, Suh MC. 2016**. Molecular and biochemical characterizations of the monoacylglycerol lipase gene family of Arabidopsis thaliana. *The Plant Journal*, **85**: 758–771.
10. **Lippold F, vom Dorp K, Abraham M, Hölzl G, Wewer V, Yilmaz JL, Lager Iì, Montandon C, Besagni C, Kessler F, Stymne S, Dörmann P.** **2012**. Fatty acid phytyl ester synthesis in chloroplasts of Arabidopsis. *The Plant Cell*, **24**: 2001–2014.
11. **Friso G, Giacomelli L, Ytterberg AJ, Peltier JB, Rudella A, Sun Q,Wijk, KJ. 2004**. In-depth analysis of the thylakoid membrane proteome of Arabidopsis thaliana chloroplasts: new proteins, new functions, and a plastid proteome database. *The Plant Cell*, **16**: 478–499.
12. **Jones MA, Morohashi K, Grotewold E, Harmer SL. 2019.** Arabidopsis JMJD5/JMJ30 Acts Independently of LUX ARRHYTHMO within the plant circadian clock to enable temperature compensation. *Frontiers in Plant Science*, **10**: 57.
13. **Eastmond PJ.** **2007**. MONODEHYROASCORBATE REDUCTASE4 is required for seed storage oil hydrolysis and postgerminative growth in Arabidopsis. *The Plant Cell*, **19**: 1376–1387.
14. **Pei H, Ma N, Tian J, Luo J, Chen J, Li J, Zheng Y, Chen X, Fei Z, Gao J. 2013.** An NAC transcription factor controls ethylene-regulated cell expansion in flower petals. *Plant Physiology*, **163**: 775–791.
15. **Zhang X, Han X, Shi R, Yang G, Qi L, Wang R, Li G. 2013.** Arabidopsis cysteine-rich receptor-like kinase 45 positively regulates disease resistance to Pseudomonas syringae. *Plant Physiology and Biochemistry,* **73**, 383–391.
16. **Thomas SG, Phillips AL, Hedden P. 1999.** Molecular cloning and functional expression of gibberellin 2- oxidases, multifunctional enzymes involved in gibberellin deactivation. *Proceedings of the National Academy of Sciences of the United States of America*, **96**: 4698–4703.
17. **Seok HY, Lee SY, Sarker S, Bayzid M, Moon YH.** **2023**. Genome-wide analysis of stress-responsive genes and alternative splice variants in *Arabidopsis* roots under osmotic stresses. International Journal of Molecular Sciences. **24**:14580.
18. **Wu J, Yan M, Zhang D, Zhou D, Yamaguchi N, Ito T. 2020**. Histone demethylases coordinate the antagonistic interaction between abscisic acid and brassinosteroid signaling in *Arabidopsis*. *Frontiers in Plant Science*, **11**: 596835.
19. **Vlot AC, Liu PP, Cameron RK, Park SW, Yang Y, Kumar D, Zhou F, Padukkavidana T, Gustafsson C, Pichersky E, Klessig DF. 2008.** Identification of likely orthologs of tobacco salicylic acid-binding protein 2 and their role in systemic acquired resistance in Arabidopsis thaliana. *The Plant Journal*, **56**: 445–456.
20. **Yang Y, Xu R, Ma CJ, Vlot AC, Klessig DF, Pichersky E. 2008.** Inactive methyl indole-3-acetic acid ester can be hydrolyzed and activated by several esterases belonging to the AtMES esterase family of Arabidopsis. *Plant Physiology*, **147**: 1034–1045.
21. **Huque AKMM, So W, Noh M, You MK, Shin J. S. 2021**. Overexpression of AtBBD1, Arabidopsis bifunctional nuclease, confers drought tolerance by enhancing the expression of regulatory genes in aba-mediated drought stress signaling. *International Journal of Molecular Sciences*, **22:** 2936.
22. **Mao G, Seebeck T, Schrenker D, Yu O. 2013**. CYP709B3, a cytochrome P450 monooxygenase gene involved in salt tolerance in Arabidopsis thaliana. *BMC Plant Biology*, **13**: 169.
