# Supplementary material for: The effect of constitutive root isoprene emission on root phenotype and physiology under control and salt stress conditions
Source: Plant Direct. 2024 Jul 6;8(7):e617. doi: 10.1002/pld3.617 (PMC11227114; doi:10.1002/pld3.617)
Supplement: Supplementary file 1 — Data S1. Peer Review. [file PLD3-8-e617-s002.pdf]

Plant Direct
Open Access

The effect of constitutive root isoprene emission on root phenotype and physiology under control and salt stress conditions

Manuel Bellucci[1,2,3]*, Mohammad Golam Mostofa[1,3,4]*, Sarathi M. Weraduwage[5], Yuan Xu[1], Mostafa Abdelrahman[6], Laura De Gara[2], Francesco Loreto[7,8], Thomas D. Sharkey[1,3,4#]

Decision Letter Round 1:

April 25, 2024

Prof. Thomas D. Sharkey
Michigan State University
Biochemistry and Molecular Biology
East Lansing, Michigan 48824

RE: The effect of constitutive root isoprene emission on root phenotype and physiology under control and salt stress conditions

Dear Dr. Sharkey:

Thank you for submitting to Plant Direct. All required reviews have been returned and we have now finished our evaluation of your manuscript. In light of the reviewers' and editor's comments, further revisions are needed before the paper can be accepted for publication in Plant Direct.

Please view the editors' and reviewers' comments below and use their suggestions as a guide while you work on your revision.

When uploading the revised version of this article, please be sure to include the following:

-A word document that contains your response to the reviewers. You should respond to each reviewer comment and note the changes made to the manuscript. If you do not agree with a reviewer's comment and choose not to make a suggested revision, please explain why. Please try to provide as complete an answer as possible to each reviewer's criticisms.
-A tracked changes document with each change highlighted
- A clean version of the latest version of the manuscript

Plant Direct

Open Access

To upload your revision, please click the link below.
https://plantdirect.msubmit.net/cgi-bin/main.plex?el=A4Lr6gv6A4Wg6I5A9ftdyABibb3m8NCNtmNXC7mieQZ

In order to provide as timely a service as possible, we ask that your revision is resubmitted within three months after receipt of this request. If an extension is needed, please send a request, along with a brief explanation, to the editorial office at plantdirect@wiley.com .

Please note that, in addition to publishing reviewer comments, the author's responses to review comments will also be published alongside the final version of the paper. If you would not like the author's responses to be published, please contact the editorial office at plantdirect@wiley.com .

Thank you very much for giving us an opportunity to review your work. I look forward to receiving the next version.

Sincerely,

Hongtao Ji

Gustavo MacIntosh

Editor, Plant Direct

-- Editor comments:

-- Reviewer comments:
Reviewer #1:

I acknowledge the effort of authors to explicitly characterize root phenotypes of isoprene-emitting transgenic lines in Arabidopsis using broad range of analyses including metabolomic and transcriptomic. There is clear effect of isoprene emissions on root phenotypes. Below I express my concerns about the manuscript.

1. Some analyses are performed with missing information and inadequate detail:
In the methods section, especially analyses on metabolites, there is no description of how they are being quantified, and if there is a normalization to input samples (dry or fresh weight), to total extracted compounds.
Analyses resulted in figure 5 and 6, including PCA analysis, t-distributed stochastic neighbour embedding, Heatmap clustering were not described in the method section.
There is a lack of description of genes that are input for all these above mentioned analyses, which makes the interpretation of result questionable.
What is the rational of transcriptome using samples collected after 4 hours of salt treatments while phenotypes were collected days after.
Authors proposed a list of candidate genes (Table S2), however it was not clear the origin of these genes, from which comparison or overlapped gene list.
On line 244, authors stated the number of core genes up and down regulated in only IE lines. These numbers are the sum of genes that are unique to each of IE line and the overlapped between 2 IE lines. The core response genes that are specific to IE lines and represent the cost of emitting isoprene should only be the number uniquely and overlapped between the 2 IE lines, that means 33 up-regulated and 49 down-regulated. The other genes that present only in one of the IE line could be a result of the background of that transformed line and cannot justify the effect of isoprene. Could author provide their rational to take those number stated in the manuscript?
2. Conclusions and messages of the results were mostly descriptive giving a general impression of what happens in the root tissue without substantiation of take-home messages.
In the previous study Zuo et al., 2019, where these IE lines were generated, transcriptome was performed for above ground tissues. The authors could have seen if what was observed in transcriptome data of root tissues if there are any tissue specific pattern, or that is a systemic response.
There are studies on salinity response with transcriptome analysis, although the focus of this manuscript not really on the effect of salt but the interaction of isoprene and salt. Author could also check if there is any special pathways that effected by isoprene in respect to salt response.
The IE lines contain a rubisco promoter, which expresses mainly on rosette, and hardly in roots. How could they explain the fact the IE lines emit isoprene via root?
There is analysis using plate rotation, there is a phenotype, but there was no discussion on how such a phenotype is linked to other phenotypes.
Isoprene emission is costly, but results of Fig 1 shows that there is a higher root growth in IE lines while the exact same IE lines had also higher above ground tissue growth (Zuo et al., 2019). Could author hypothesize the explanation for this? What might be the trait-off?
3. Other revisions to be taken care
Line 127 PR growth: primary root growth?
Legend of Fig. S2 shows treatment 150mM, in the text stated 100mM

Please check legend of other Supp. Figures, sometimes they miss the narration of which one is (a), which one is (b) precisely. E.g Fig S1
Line 144 LR: lateral root?
Line 152-153: ""not statistically significant in line C4, Fig.2a", but in fact it is significant
Line 162: Author uses NE here, however it needs to be precise if it is EV or wildtype. Only use NE if that represents both EV and wildtype at the same time.
Line 166: "MeJA but not JA-Ile significantly decreased", according to the Fig. 3 it is not correct
Line 177: "all cytokinins remained significantly elevated in IE lines under salt stress (Fig. 3g,h,i)." That is not correct, the trends are not consistent between 2 transgenic lines when comparing salt treatment and control. Only the level of all cytokinins in IE lines were higher than EV lines.
The submitted version includes 2 times the text including tracking. Is there a technical issue during submission?

Reviewer #2:

This paper by Bellucci et al. examines the effect of isoprene emission on root phenotype and physiology under different salinity stresses. Overall, this is mainly a clear and well written manuscript. There are some minor issues (detailed below) but there is a clear narrative. Each of the sub-sections within the methods section describes the protocols used clearly and concisely, and in sufficient detail that these can be replicated. Where some details are truncated, they refer to established peer-reviewed protocols instead. In the results section, the descriptions of the data are consistent with the Figures and Tables.
The study is well designed to test the aims detailed in its introduction and it describes a wide range of analyses, all of which are entirely appropriate for what is being measured. The main detail that I found missing from the description of the experiments was the number of replicates used in the different elements of the experimental design. These details only became apparent after reading the figure legends, so these details need including in the main body of the text, either in their respective sub-section of the methods or under the statistical analysis section. This is important because in the statistics section, a two-way ANOVA is described for examining differences among the different lines and salinity treatments for several variables. For many of these variables, they were examined using 4 plates with 12 plants on each plate, but there was no mention of ensuring n=4 in the statistical analyses because the plates are the replicates in the described design, not the plants. If plants were separated out and grown individually on solid media and I've misinterpreted this, then the description in the methods needs to make this clear. If not, and the 12 plants were kept on one plate, and the authors used "plate" means in the ANOVA instead to account for this, reducing the likelihood of a type I error, then please state this. If not, I would suggest that "plate" be used as a random factor in a linear

mixed model analysis as an alternative statistical approach. So, although I have no concerns with the trends and patterns that the authors show, I would like some reassurance on the robustness of some of their statistical conclusions.

Minor comments:
Line 127: PR is not defined prior to that point (Please include descriptor).
Line 130: a non-significant reduction effectively means no reduction, so just say that.
Line 507-508: You state you use water and acetonitrile as the mobile phase. Please state the proportions if run isocratically or specify the gradient program used.
Fig.1a,b,c: The scale bar needs a value on it. I know it's described in the legend, but it needs including in the image too. Also, is the width of the orange bar the same scale as the scale bar?
Figure 6c: alpha-linolenic acid metabolism shows as being both up- and down-regulated in the KEGG analysis. But this doesn't align with what you described in the results. Is this a labelling error or does this need explaining in the results section?
Supplementary figures (particularly those that are boxplots): In many of the plots you state the boxplot is median with quartiles then state that all data are means and standard deviations. This needs clarification or rectification.
Figure S1: The white mark on the image being used as a scale needs the values on it.
Figure S6: This is labelled as absolute growth of primary root. However, on day Zero you have 2 cm of growth. So, are you illustrating absolute root length instead? Also, what are parts (a), (b) and (c)? There are no descriptors for them.
Figures S7 and S8: What do the scales indicate? These are not described in the legends.
Table S1: I suggest using an asterisk in the Table and Table legend to highlight pyruvate analysis was with TBDMS.
Tables S2 and S3: Why are certain genes in bold. There is no explanation.

Decision Letter Round 2:

June 2, 2024
Prof. Thomas D. Sharkey
Michigan State University
Biochemistry and Molecular Biology
East Lansing, Michigan 48824

MSID: 2024-01462-TW1
MS TITLE: The effect of constitutive root isoprene emission on root phenotype and physiology under control and salt stress conditions

Dear Dr. Thomas Sharkey:

I am pleased to inform you that your manuscript "The effect of constitutive root isoprene emission on root phenotype and physiology under control and salt stress conditions" has been accepted for publication in Plant Direct.

Your article will appear online in the next available issue of Plant Direct. To ensure your article gets published as quickly as possible, please pay attention to the steps detailed below. We have found that most of the delays happen at this stage, especially at the payment stage, so please respond as quickly as possible when prompted.

License Agreement: Once your article has been accepted it will move to Production and undergo admin and file checking - you may receive an email with any queries we have at this stage. When all required items are received by the publisher and queries resolved, the corresponding author will receive an email from Wiley's Author Services system which will ask them to log on at https://authorservices.wiley.com/bauthor and will present them with the appropriate license for completion. Your article cannot be published until both the signed license agreement and payment of the article fee have been received.

Payment of the Open Access Article Publication Fee: All articles published in Plant Direct are fully open access: immediately and freely available to read, download and share. Plant Direct charges a publication fee to cover the publication costs. The corresponding author for this manuscript should have already received a quote from the payments team (cs-openaccess@wiley.com) with the estimated article publication fee; please email cs-openaccess@wiley.com if this has not been received. The corresponding author should log on to the Wiley Author Services site, where the publication fee can be paid by credit card or an invoice. Pro Forma can also be requested. Payment of the publication charge must be received before the article will be published online.

Proofs: You will have the opportunity to look over your paper once more when you receive the author proofs for your article. The proofs will be with you in approximately two weeks. Please note that, in addition to publishing reviewer comments, the author's responses to review comments will also be published alongside the final version of the paper. If you would not like the author's responses to be published, please contact the editorial office at plantdirect@wiley.com .

Promotion of your article: You can help your research get the attention it deserves! Check out Wiley's free Promotion Guide for best-practice recommendations for promoting your work at www.wileyauthors.com/eeo/guide. And learn more about Wiley Editing Services which offers professional video, design, and writing services to create shareable video abstracts,

infographics, conference posters, lay summaries, and research news stories for your research at www.wileyauthors.com/eeo/promotion.

Thank you again for your contribution to Plant Direct. If you have any questions, feel free to contact the editorial office at plantdirect@wiley.com .

Sincerely,

Hongtao Ji

Gustavo MacIntosh

Editor, Plant Direct

Below are some minor points to help authors improve their manuscript further.

-- Editor comments

Please check the specification of your manuscript carefully. Make sure that there are no typing errors, genes are written in italics and that there is consistency of expression throughout the text, et al.

-- Reviewer comments:
Reviewer #1:

Authors have addressed properly my points of concern.

I only wonder why phenotypic analysis was performed on plants under up to 100 mM NaCl treatment, while analytical analyses (metabolome and transcriptome) used samples of 150 mM NaCl. Could authors react on this?

There is still formatting consistency issues, please revisit throughout the manuscript
Abbreviation of gene names: L80 PcIspS, other cases L432 EgISPS in capital letter
L126 and other places: 2-wk-old while should be two-week-old to be consistent with five-day-old seedlings in L146
L190 Fig. 4a-d while in L192-193 Fig. 4 a,b,c,d instead
Table S4. Genes that were differentially expressed in B2 and C4: in is missing. Also some genes

are not in italic letters while others are.

Reviewer #2:

The revised manuscript of Bellucci et al. remains a well written paper with a clear narrative even with the revisions asked for by both original reviewers. If anything, the revisions make the story even more straightforward to understand. Hence, I am satisfied that the authors have addressed the original reviewers' concerns, provided the extra details where requested, and made the necessary alterations to text and figures where required.

Author's Response:

Reviewer #1:

I acknowledge the effort of authors to explicitly characterize root phenotypes of isoprene-emitting transgenic lines in Arabidopsis using broad range of analyses including metabolomic and transcriptomic. There is clear effect of isoprene emissions on root phenotypes. Below I express my concerns about the manuscript.

We acknowledge your positive evaluation of our manuscript. We also thank the reviewer for the constructive suggestions. Following your advice, we have modified our manuscript as you can see our point-by-point responses to your comments below.

Comment 1

Some analyses are performed with missing information and inadequate detail:
In the methods section, especially analyses on metabolites, there is no description of how they are being quantified, and if there is a normalization to input samples (dry or fresh weight), to total extracted compounds.

Response

We have used fresh weight (FW) to quantify the levels of all the metabolites. We have revised the method sections accordingly. (lines 631; 653; 656)

Comment 2

Analyses resulted in figure 5 and 6, including PCA analysis, t-distributed stochastic neighbour embedding, Heatmap clustering were not described in the method section. There is a lack of description of genes that are input for all these above mentioned analyses, which makes the interpretation of result questionable.

Response

We have revised the Materials and methods to include detailed descriptions of all the methods for analyzing the data obtained from the current study.

"For normalization of the read counts, we applied a variance stabilizing transformation (VST). This approach was used to stabilize the variance across our dataset. Subsequently, the 6,000 most variable genes were identified and selected for further analysis. These genes were chosen due to their high variability among the different genotypes, indicating their potential biological significance. We utilized this subset of genes for constructing heatmap clustering, principal component analysis (PCA), and t-SNE to explore and visualize the data structure and relationships using iDEP 0.96 bioinformatic tool (http://bioinformatics.sdstate.edu/idep96/)."

Comment 3

What is the rational of transcriptome using samples collected after 4 hours of salt treatments while phenotypes were collected days after.

Response

It is well known that transcripts are affected earlier than metabolites, and phenomes. Moreover, measurements were made to obtain enough material to do the analysis. Five days of salt exposure would have significantly reduced root growth (especially in EV).

Comment 4

Authors proposed a list of candidate genes (Table S2), however it was not clear the origin of these genes, from which comparison or overlapped gene list.

Response

The genes shown in the tables come from the list of genes that are upregulated and downregulated (when exposed to salt) in both the isoprene lines (B2 and C4 lines) compared to both NE lines (EV and WT).

Comment 5

On line 244, authors stated the number of core genes up and down regulated in only IE lines. These numbers are the sum of genes that are unique to each of IE line and the overlapped between 2 IE lines. The core response genes that are specific to IE lines and represent the cost of emitting isoprene should only be the number uniquely and overlapped between the 2 IE lines, that means 33 up-regulated and 49 down-regulated. The other genes that present only in one of the IE line could be a result of the background of that transformed line and cannot justify the effect of isoprene. Could author provide their rational to take those number stated in the manuscript?

Response

We would like to thank the Reviewer for his/her insightful comments regarding the categorization of core response genes in isoprene-emitting (IE) lines. We acknowledge the importance of distinguishing between genes that are uniquely affected by isoprene emission and those that might be influenced by other genetic background factors in the transformed lines. In our manuscript, we reported numbers of up-regulated and down-regulated genes by combining those **UNIQUE** to each IE line and those **OVERLAPPING** between the **TWO** IE lines. We want to clarify that our analytical approach specifically focused on **comparing IE lines** under salt stress versus **IE lines** under non-stress conditions (B2150_vs_B2, and C4150_vs_C4). By doing this, we deliberately excluded comparisons that could introduce **background effects** or **NON-IE**, such as EV10_vs_EV, and WT150_vs_WT. Thus, the background effect due to

transformation in each IE line was minimized. We revised the result section accordingly as indicated below:

"In contrast, 373 (186, 33 and 154) up-regulated genes and 415 (175, 49 and 191) down-regulated genes were identified only in IE lines under control and salt stress conditions (B2150_vs_B2, and C4150_vs_C4). (Fig. 6b). By doing this, we deliberately excluded comparisons that could introduce background or NON-IE effects, such as EV10_vs_EV, and WT150_vs_WT. These 788 (373 and 415) …"

Comment 6

Conclusions and messages of the results were mostly descriptive giving a general impression of what happens in the root tissue without substantiation of take-home messages.

Response

In the conclusion, we have provided information on how a tiny emission of isoprene from roots could lead to the improvement of root growth and salt resistance, which is our main take-home message from this study. We have also mentioned future perspectives from the current findings.

Comment 7

In the previous study Zuo et al., 2019, where these IE lines were generated, transcriptome was performed for above ground tissues. The authors could have seen if what was observed in transcriptome data of root tissues if there are any tissue specific pattern, or that is a systemic response.

Response

We appreciate the Reviewer's suggestion to compare the transcriptomic data between above ground and root tissues. In the referenced study by Zuo et al., 2019, where the isoprene-emitting (IE) lines were initially characterized, transcriptomic analysis was indeed performed on above ground tissues. This provided initial insights into the systemic responses and tissue-specific patterns of gene expression related to isoprene biosynthesis. In this study, we were focusing on the salinity stress responses in the IE lines and non-IE lines as main objective.

Nevertheless, we conducted a comparative analysis of isoprene biosynthesis-related genes expressed in root tissues against those previously reported for above ground tissues. This comparison aimed to identify any unique or shared regulatory patterns that could signify tissue-specific responses or indicate a systemic adaptation mechanism within the plant.

Comment 8

There are studies on salinity response with transcriptome analysis, although the focus of this manuscript not really on the effect of salt but the interaction of isoprene and salt. Author could also check if there is any special pathways that effected by isoprene in respect to salt response.

Response

In this manuscript, we have mainly focused on the effects of isoprene on root development under salt stress. Given the role of isoprene as signaling molecule, we agree with the reviewer that isoprene might have modulated salt tolerance mechanisms in Arabidopsis, which we will dig into details in our further studies.

Comment 9

The IE lines contain a rubisco promoter, which expresses mainly on rosette, and hardly in roots. How could they explain the fact the IE lines emit isoprene via root?

Response

Expression of Arabidopsis rbcs1 promoter gene has also been reported in the non-photosynthetic organs like root tip (please see the reference below). Our study revealed a tiny level of root isoprene in both transgenic lines overexpressing *ISPS* under the promoter rbcs1. We also do not rule out the possibility of translocation of isoprene from aboveground to belowground, which perhaps also contributed to root isoprene emission.

Reference

1. Megan G. Sawchuk, Tyler J. Donner, Philip Head, Enrico Scarpella, Unique and Overlapping Expression Patterns among Members of Photosynthesis-Associated Nuclear Gene Families in Arabidopsis, *Plant Physiology*, Volume 148, Issue 4, December 2008, Pages 1908–1924

Comment 10

There is analysis using plate rotation, there is a phenotype, but there was no discussion on how such a phenotype is linked to other phenotypes.

Response

We have added a discussion on the benefits of root gravitropism under saline conditions (lines 403-406).

Comment 11

Isoprene emission is costly, but results of Fig 1 shows that there is a higher root growth in IE lines while the exact same IE lines had also higher above ground tissue growth (Zuo et al., 2019). Could author hypothesize the explanation for this? What might be the trait-off?

Response

We clearly show a much higher root/shoot ratio in IE, which suggests that higher root growth goes at the expense of aboveground growth. Although the growth conditions were different, our results are in contrast from those shown by Zuo et al., 2019. We highlighted this difference in the main text (lines 205-206)

3. Other revisions to be taken care

Comment 12

Line 127 PR growth: primary root growth?

Response

Yes, PR means primary root, which was already abbreviated before (line 182)

Comment 13

Legend of Fig. S2 shows treatment 150 mM, in the text stated 100 mM

Response

It should be 100 mM NaCl. We have corrected the sentence in the legend.

Comment 14

Please check legend of other Supp. Figures, sometimes they miss the narration of which one is (a), which one is (b) precisely. E.g Fig S1

Response

We have revised and clearly mentioned the identity of each panel in the legend.

Comment 15

Line 144 LR: lateral root?

Response

Yes, LR means lateral root which was clarified.

Comment 16

Line 152-153: ""not statistically significant in line C4, Fig.2a", but in fact it is significant

Response

We have revised and corrected the sentence (lines 219-220)

Comment 17

Line 162: Author uses NE here, however it needs to be precise if it is EV or wildtype. Only use NE if that represents both EV and wildtype at the same time.

Response

We have followed this suggestion throughout the manuscript.

Comment 18

Line 166: "MeJA but not JA-Ile significantly decreased", according to the Fig. 3 it is not correct

Response

We have corrected the sentence in the revised manuscript.

Comment 19

Line 177: "all cytokinins remained significantly elevated in IE lines under salt stress (Fig. 3g,h,i)." That is not correct, the trends are not consistent between 2 transgenic lines when comparing salt treatment and control. Only the level of all cytokinins in IE lines were higher than EV lines.

Response

We have corrected and revised the results accordingly.

Comment 20

The submitted version includes 2 times the text including tracking. Is there a technical issue during submission?

Response

Yes, this is probably a technical issue

Reviewer #2:

This paper by Bellucci et al. examines the effect of isoprene emission on root phenotype and physiology under different salinity stresses. Overall, this is mainly a clear and well written manuscript. There are some minor issues (detailed below) but there is a clear narrative. Each of the sub-sections within the methods section describes the protocols used clearly and concisely, and in sufficient detail that these can be replicated. Where some details are truncated, they refer to established peer-reviewed protocols instead. In the results section, the descriptions of the data are consistent with the Figures and Tables. The study is well designed to test the aims

detailed in its introduction and it describes a wide range of analyses, all of which are entirely appropriate for what is being measured.

Response

Thank you very much for evaluating our manuscript and providing constructive suggestions. We followed your suggestions and revised the manuscript as you can see our responses to your comments below.

Comment 1

The main detail that I found missing from the description of the experiments was the number of replicates used in the different elements of the experimental design. These details only became apparent after reading the figure legends, so these details need including in the main body of the text, either in their respective sub-section of the methods or under the statistical analysis section. This is important because in the statistics section, a two-way ANOVA is described for examining differences among the different lines and salinity treatments for several variables. For many of these variables, they were examined using 4 plates with 12 plants on each plate, but there was no mention of ensuring n=4 in the statistical analyses because the plates are the replicates in the described design, not the plants. If plants were separated out and grown individually on solid media and I've misinterpreted this, then the description in the methods needs to make this clear. If not, and the 12 plants were kept on one plate, and the authors used "plate" means in the ANOVA instead to account for this, reducing the likelihood of a type I error, then please state this. If not, I would suggest that "plate" be used as a random factor in a linear mixed model analysis as an alternative statistical approach. So, although I have no concerns with the trends and patterns that the authors show, I would like some reassurance on the robustness of some of their statistical conclusions.

Response

We have provided details of the number of replicates and plants for individual experiment in the respective sub-section of the main text and in the figure legends. (line 609-703)

Minor comments:

Comment 2

Line 127: PR is not defined prior to that point (Please include descriptor).

Response

In the current manuscript, we have abbreviated PR as primary root at its first appearance.

Comment 3

Line 130: a non-significant reduction effectively means no reduction, so just say that.

Response

We have revised the sentence accordingly (line 191)

Comment 4

Line 507-508: You state you use water and acetonitrile as the mobile phase. Please state the proportions if run isocratically or specify the gradient program used.

Response

We have provided gradient sequence information in the Table S2 and Table S3 of the current version of the manuscript.

Comment 5

Fig.1a,b,c: The scale bar needs a value on it. I know it's described in the legend, but it needs including in the image too. Also, is the width of the orange bar the same scale as the scale bar?

Response

We have added the value to the scale bar in the figure. The orange lines are used to indicate the different length of the roots across the treatments.

Comment 6

Figure 6c: alpha-linolenic acid metabolism shows as being both up- and down-regulated in the KEGG analysis. But this doesn't align with what you described in the results. Is this a labelling error or does this need explaining in the results section?

Response

Thank you for your query regarding the depiction of alpha-linolenic acid metabolism in Figure 6c of our manuscript. We appreciate your attention to detail in noting the apparent contradiction between the up- and down-regulation shown in the KEGG analysis, and the description provided in the Results section. This observation in the figure is indeed correct and not a labeling error. The alpha-linolenic acid pathway was found to exhibit both up- and down-regulation of different genes within the same metabolic pathway. This dual regulation reflects the complex nature of metabolic responses to stress conditions, where some enzymes may be up-regulated to enhance specific branches of this pathway, while others are down-regulated to suppress alternate branches or conserve energy and resources. Nevertheless, the number of fatty acid-related pathways were more enriched in the up-regulated genes compared with down-regulated one. We revised the result section accordingly as indicated in lines 379-381.

Comment 7

Supplementary figures (particularly those that are boxplots): In many of the plots you state the boxplot is median with quartiles then state that all data are means and standard deviations. This needs clarification or rectification.

Response

We have revised and provided much information for easy understanding of all the boxplots in the current manuscript (L.).

Comment 8

Figure S1: The white mark on the image being used as a scale needs the values on it.

Response

We have added the value for the scale bar.

Comment 9

Figure S6: This is labelled as absolute growth of primary root. However, on day Zero you have 2 cm of growth. So, are you illustrating absolute root length instead? Also, what are parts (a), (b) and (c)? There are no descriptors for them.

Response

We have revised the legend accordingly. The data are recorded at different time points (days 0 to 5) after exposure of five-day-old seedlings to different concentrations of salt (0, 50, and 100 mM NaCl).

Comment 10

Figures S7 and S8: What do the scales indicate? These are not described in the legends.

Response

We have revised and clarified the scale bars in both figures.

Comment 11

Table S1: I suggest using an asterisk in the Table and Table legend to highlight pyruvate analysis was with TBDMS.

Response

We have revised accordingly.

Comment 12

Tables S2 and S3: Why are certain genes in bold. There is no explanation.

Response

Bold genes are upregulated genes. We have explained this in the legends of Tables S4 and S5 of the revised manuscript.

****
